# Evolutionary diversification of retinoic acid receptor ligand-binding pocket structure by molecular tinkering

**DOI:** 10.1098/rsos.150484

**Published:** 2016-03-16

**Authors:** Juliana Gutierrez-Mazariegos, Eswar Kumar Nadendla, Romain A. Studer, Susana Alvarez, Angel R. de Lera, Shigehiro Kuraku, William Bourguet, Michael Schubert, Vincent Laudet

**Affiliations:** 1 Molecular Zoology Team, Institut de Génomique Fonctionnelle de Lyon, Université de Lyon, Université Lyon 1, CNRS, INRA, Ecole Normale Supérieure de Lyon, 46 Allée d'Italie, 69364 Lyon Cedex 07, France; 2 Centre de Biochimie Structurale, Inserm U1054, CNRS UMR 5048, Université de Montpellier, 29 Rue de Navacelles, 34090 Montpellier, France; 3 European Molecular Biology Laboratory, European Bioinformatics Institute, (EMBL-EBI)—Wellcome Genome Campus, Hinxton, Cambridge CB10 1SD, UK; 4 Departamento de Química Organica, Facultad de Química, Universidade de Vigo, 36310 Vigo, Spain; 5 Phyloinformatics Unit, RIKEN Center for Life Science Technologies, 2-2-3 Minatojima-minamimachi, Chuo-ku, Kobe, Hyogo 650-0047, Japan; 6 Sorbonne Universités, UPMC Université Paris 06, CNRS, UMR 7009, Laboratoire de Biologie du Développement de Villefranche-sur-Mer, Observatoire Océanologique de Villefranche-sur-Mer, 181 Chemin du Lazaret, 06230 Villefranche-sur-Mer, France

**Keywords:** cyclostomes, emergence of evolutionary novelty, gene and whole genome duplication, hagfish and lamprey, nuclear hormone receptor signalling, vertebrate evolution

## Abstract

Whole genome duplications (WGDs) have been classically associated with the origin of evolutionary novelties and the so-called duplication–degeneration–complementation model describes the possible fates of genes after duplication. However, how sequence divergence effectively allows functional changes between gene duplicates is still unclear. In the vertebrate lineage, two rounds of WGDs took place, giving rise to paralogous gene copies observed for many gene families. For the retinoic acid receptors (RARs), for example, which are members of the nuclear hormone receptor (NR) superfamily, a unique ancestral gene has been duplicated resulting in three vertebrate paralogues: RARα, RARβ and RARγ. It has previously been shown that this single ancestral RAR was neofunctionalized to give rise to a larger substrate specificity range in the RARs of extant jawed vertebrates (also called gnathostomes). To understand RAR diversification, the members of the cyclostomes (lamprey and hagfish), jawless vertebrates representing the extant sister group of gnathostomes, provide an intermediate situation and thus allow the characterization of the evolutionary steps that shaped RAR ligand-binding properties following the WGDs. In this study, we assessed the ligand-binding specificity of cyclostome RARs and found that their ligand-binding pockets resemble those of gnathostome RARα and RARβ. In contrast, none of the cyclostome receptors studied showed any RARγ-like specificity. Together, our results suggest that cyclostome RARs cover only a portion of the specificity repertoire of the ancestral gnathostome RARs and indicate that the establishment of ligand-binding specificity was a stepwise event. This iterative process thus provides a rare example for the diversification of receptor–ligand interactions of NRs following WGDs.

## 1. Background

In the 1970s, Ohno [1] proposed that gene duplication was an important mechanism to produce evolutionary novelties. Later on, it was effectively shown that two rounds (2R) of whole genome duplications (WGDs) occurred early during vertebrate evolution [2–4]. Although this idea was initially very controversially discussed, the existence of these two WGDs is now well accepted, and this is mainly due to the unique datasets provided by the sequencing of the complete genomes of various chordate species [3,5–7]. Evidence for these ancient duplication events can be found in vertebrate genomes, even if only some duplicated genes (called ‘ohnologues’) organized in ‘paralogons’ have been retained in extant vertebrate species [8]. In contrast, the timing of the two rounds of WGD in vertebrates relative to the main phylogenetic events, that is the emergence of the different vertebrate groups, has been difficult to determine. Although it has already been established a while ago that the two WGDs occurred at the base of vertebrates, before the emergence of the chondrichthyans [9–11], the question whether the genome duplications occurred before or after the cyclostome–gnathostome split remains contested [7,12,13]. The resolution of this problem has been obscured both by lineage-specific gene duplications, at least in the lamprey lineage [14,15], as well as by dramatic genomic rearrangement programmed in the genomes of somatic cell lineages in both lamprey and hagfish [16–19]. Nonetheless, recent analyses have provided support for the notion that the two WGDs occurred before the cyclostome–gnathostome split, indicating that cyclostomes are characterized by genomes that are as complex as the ones of gnathostomes [19–21].

The timing of the WGDs at the base of the vertebrate lineage is intimately linked to another controversial issue related to gene duplications as a whole, which is the actual link of gene duplications to the emergence of evolutionary novelties [2,4,22]. Indeed, the question of the origin of novelties and their relation to genomic events is central in evolutionary biology and has been much discussed [3,4,23,24]. The occurrence of WGDs should, in principle, provide the potential for functional innovation by divergence of ohnologues. A general model, called the duplication–degeneration-complementation (DDC) model, has been proposed to describe the various possible fates of genes after duplication (non-functionalization, subfunctionalization or neofunctionalization) [25,26]. Strikingly, very few functional novelties created by gene neofunctionalization have been demonstrated to date and this lack of experimental support for the DDC model, therefore, remains an important issue to tackle [3,4]. However, many cases of duplicated genes with evolutionary patterns of neofunctionalization have been identified [27–29], including several nuclear hormone receptors (NRs) [30].

NRs are a phylogenetically related superfamily of ligand-dependent transcription factors that regulate many biological functions, including embryonic development, metamorphosis, metabolism and homeostasis [31]. They represent a very good model to investigate the functional role of WGDs in organismal evolution, because they are dispersed in the genome, allow robust phylogenetic reconstruction and are functionally well characterized, with specific functional adaptations having been described for different NR paralogues [32–35]. Among the NRs, the retinoic acid receptors (RARs) are particularly interesting, because RARs control key steps in embryonic development and thus provide the possibility to establish testable hypotheses linking gene duplications to the appearance of functional novelties [36].

In mammals, there are three RAR paralogues, RARα, RARβ and RARγ, that were shown to be products of the vertebrate-specific WGDs [12,33]. Like other NRs, RARs have a modular structure with two major conserved domains, the DNA-binding domain (DBD) and the ligand-binding domain (LBD) [37]. The LBD mediates the binding to the ligand and is composed of 12 α-helices (H1–H12) and a. Of these 12 α-helices, H12 is particularly important for controlling the transcriptional activity of the receptor, as its position is modified by the binding of the ligand. The ligand binds to a hydrophobic pocket, the so-called ligand-binding pocket (LBP), formed by 25 residues inside the LBD, which are localized in H1, H3, H5, the β-turn, loop 6–7, H11, loop 11-12 and H12 itself. Only one *in vivo* ligand has been identified for vertebrate RARs, all-*trans* retinoic acid (ATRA), and genetic evidence in mice has suggested that other retinoic acid metabolites do not play a significant role during development [38]. However, the LBPs of human RARs differ from each other in three amino acid positions (serine 232, isoleucine 270 and valine 395 in H3, H5 and H11 of human RARα, respectively) [39,40], and these differences cause alterations in the binding specificities of the RAR paralogues *in vitro*, which explains the different pharmacology exhibited by each of the three paralogues [41]. Furthermore, although it is not known whether these ligand specificity differences have functional roles *in vivo*, synthetic retinoids have been developed that can bind to and trigger the transactivation of individual RAR paralogues [42].

Because ligand-binding specificity and LBP structure are directly correlated, we previously used paralogue-specific synthetic retinoids as markers for the LBP structure of RARs. Comparisons of ligand-binding abilities of different RARs from organisms located at key phylogenetic positions and of RARs with mutated LBPs hence provided novel insights into the evolution of LBP structure and function and allowed us to hypothesize that, whereas RARβ kept an ancestral LBP conformation, RARα and RARγ diverged in ligand-binding capacity. Our work further suggested that neofunctionalization occurred, not only at the functional level to shape the roles of RARs in vertebrates, but also on the gene regulatory level leading to alterations in developmental gene expression [33].

As discussed above, the evolutionary history of WGDs in early vertebrates is quite complex, as the WGDs were followed by massive gene losses and the divergence of the duplicated copies retained in the genome. To better understand the relationship between WGDs and the functional diversification of duplicated genes in vertebrates, the cyclostomes are of particular interest as starting point for comparative studies. Owing to their phylogenetic position as a sister group of gnathostomes [14], cyclostomes can be used to reveal insights into the mechanisms of neofunctionalization following the vertebrate WGDs, for example, whether this process occurred rapidly or slowly after the duplications, at once or in several steps. To date, three RARs, called RAR1, RAR2 and RAR3 have been described in the inshore hagfish *Eptatretus burgeri* and four RARs, called RAR1, RAR2, RAR3 and RAR4, have been identified in three different lamprey species: the Japanese lamprey *Lethenteron japonicum*, the European freshwater lamprey *Lampetra fluviatilis* and the sea lamprey *Petromyzon marinus* [20,43]. Importantly, phylogenetic analyses suggest that lamprey RAR1 and RAR4 are the result of a lamprey-specific duplication event that took place after the hagfish–lamprey split [43]. Although the developmental gene expression has been described at least for the lamprey RARs, the functions of the cyclostome receptors in embryo and adult still remain elusive [43]. Furthermore, it has previously been shown that one of the RARs from the sea lamprey *P. marinus*, now designated RAR4, has a ligand-binding specificity similar to that of gnathostome RARα and RARβ, but nothing is known about the pharmacology of the other cyclostome RARs [33].

Here, we describe the molecular characterization of the three RARs (RAR1, RAR2 and RAR3) from the hagfish *E. burgeri* and of three of the four RARs (RAR1, RAR2 and RAR3) from the lamprey *L. japonicum*. We analysed the ability of these receptors to bind paralogue-selective compounds and to activate their transcription upon binding. We found that the studied cyclostome receptors have LBP structures that are most similar to mammalian RARα and RARβ. In addition, we reconstructed the sequence of an ancestral vertebrate RAR, which, contrary to previous reports, led us to conclude that the ancestor of all vertebrate RARs was RARα-like. The LBP structures of RARβ and RARγ were thus acquired secondarily in the gnathostome lineage. Taken together, our results demonstrate that the acquisition of the ligand-binding repertoire of vertebrate RARs was a stepwise process and provide an example for the functional evolution of gene paralogues following WGD.

## 2. Material and methods

### 2.1. Cloning of receptors and constructs

The LBD of the receptors was fused to the GAL4 DNA binding domain (amino acids 1–147) by cloning the LBD into the PG4MpolyII vector [44].

### 2.2. Sequence analysis

The accession numbers of the RAR proteins used to compile the sequence alignment are as follows: human RARα (P10276.2), RARβ (P10826.2) and RARγ (P13631.1); spotted gar (*Lepisosteus oculatus*) RARα (BAH03351.1), RARβ (BAH03352.1) and RARγ (BAH03353.1); small-spotted catshark (*Scyliorhinus canicula*) RARα (BAH03356.1), RARβ (BAH03357.1) and RARγ (SSC-transcript-ctg66621 from SkateBase.org); Japanese lamprey (*L. japonicum*) RAR1 (BAH03333.1), RAR2 (BAH03334.1) and RAR3 (BAH03335.1); inshore hagfish (*E. burgeri*) RAR1 (BAH03345.1), RAR2 (BAH03346.1) and RAR3 (BAH03347.1). The protein sequences were aligned with MAFFT [45] and visualized with either Jalview [46] or Seaview [47].

### 2.3. Probabilistic orthology assessment

We prepared datasets consisting of five operational taxonomic units (OTUs; electronic supplementary material, table S1): (i) only one cyclostome RAR gene; (ii) gnathostome RARα genes; (iii) gnathostome RARβ genes; (iv) gnathostome RARγ genes; and (v) the outgroup (electronic supplementary material, figure S1). By inputting each sequence dataset, including one selected cyclostome gene, we computed likelihood with Tree-Puzzle v.~5.2 for individual tree topologies specified in the user-defined trees mode [48]. We subsequently prepared the tree topology files using two different approaches: (i) all possible tree topologies using the five OTUs or (ii) focusing on only nine tree topologies that are compatible with the 1–2–4 duplication pattern predicted by the 2R WGDs in vertebrates (electronic supplementary material, table S2). Output files of Tree-Puzzle were processed with the program Consel v. 0.20 to obtain bootstrap probabilities based on resampling of estimated log-likelihood (RELL BP) [49]. With and without the assumption of the fourth paralogue generated in 2R WGDs, we added up the RELL BPs of tree topologies supporting the one-to-one orthologies of cyclostome RAR1, RAR2 and RAR3 to four (RARα, RARβ, RARγ and RARδ) or three (RARα, RARβ and RARγ) gnathostome RAR subtypes, respectively (electronic supplementary material, table S3).

### 2.4. Phylogenetic analyses and ancestral sequence reconstruction

Using BLASTp, we scanned the genomes of selected taxa for RAR sequences: six non-vertebrate deuterostomes, four cyclostomes, three chondrichthyans, three actinopterygians (the gar and two teleost fish) and nine sarcopterygians (the coelacanth and eight tetrapods). A total of 61 sequences were retrieved, providing a well-balanced taxonomic coverage (electronic supplementary material, table S4). These sequences were aligned with MAFFT v.~7.221 (with L-INS-i mode for accuracy) [45]. Amino acid residues forming the LBD were extracted in Jalview (electronic supplementary material, figure S2) [46]. A phylogenetic tree was built with PhyML (parameters: -m LG -c 4 -a e -s BEST -o tlr) [50] and rooted with Newick Utilities [51], using the acorn worm and sea urchin RAR sequences as outgroup. The resulting tree was then manually adjusted with PhyloWidget [52] to reconcile the phylogeny with the results of the probabilistic orthology assessment. The observed differences were due to the fact that the cyclostome sequences are unusually divergent, as has previously been observed [20]. After these corrections, CodeML [53] was used to perform the ancestral sequence reconstruction of the ancestral LBDs. All columns that contained more than 10% gaps were not included in the reconstruction. For each ancestral sequence, the probability of the presence of a particular amino acid at a particular position was visualized using WebLogo [54].

### 2.5. Ligands

ATRA was purchased from Sigma-Aldrich, whereas the RARα-specific BMS753, the RARβ-specific BMS641 and the RARγ-specific BMS961 were synthesized as previously described [55]. Stock solutions of the different compounds were prepared in ethanol at a concentration of 10^−2^ M.

### 2.6. Transactivation assays in human embryonic kidney 293T cell*s*

HEK 293T (human embryonic kidney) cells were maintained in Dulbecco's modified Eagle's medium (DMEM; Invitrogen by Life Technologies) supplemented with 10% foetal calf serum (Invitrogen by Life Technologies). Cells at 70% confluence were transfected using ExGen 500 (Euromedex) according to the instructions of the manufacturer with a plasmid containing the luciferase reporter gene (17M-Glob-Luc) and the CMV-β-Gal plasmid as internal control to normalize for variations of transfection efficiency. A total of 60 ng of DNA was transfected. Six hours after transfection, the medium of the cells was changed to DMEM supplemented with 10% charcoal-treated foetal calf serum containing the ligands diluted to predefined final concentrations. Twenty-four hours after the treatment, cells were lysed and assayed for luciferase activity using the luciferase assay reagent (Promega). For the lamprey RARs, the following final treatment concentrations were used: ATRA at 10^−8^ M, 10^−7^ M, 5 × 10^−7^ M and RARα-specific BMS753, RARβ-specific BMS641 and RARγ-specific BMS961 at 10^−8^, 10^−7^, 5 × 10^−7^ M. For the hagfish RARs, the following final treatment concentrations were used: for RAR1 and RAR3, ATRA at 10^−9^, 10^−8^, 5 × 10^−8^ M and RARα-specific BMS753, RARβ-specific BMS641 and RARγ-specific BMS961 at 10^−8^, 10^−7^, 10^−6^ M; for RAR2, ATRA at 10^−7^, 10^−6^, 10^−5^ M and RARα-specific BMS753, RARβ-specific BMS641 and RARγ-specific BMS961 at 10^−7^, 10^−6^, 10^−5^ M.

### 2.7. Limited proteolysis assays

The different RAR proteins were translated and labelled *in vitro* with radioactive ^35^S using the TNT coupled reticulocyte lysate system (Promega). The limited proteolysis assay experiments were performed as previously described [33], and the final concentrations tested for each retinoid ligand were 10^−7^, 10^−6^, 10^−5^ M.

### 2.8. Three-dimensional modelling of retinoic acid receptor ligand-binding domains

Three-dimensional models of the lamprey and hagfish RAR LBDs were constructed using the modelling meta-server @TOME 2 [56] and the crystal structures of the LBDs of human RARα (PDB code 3KMR) [57] or human RARβ (PDB code 4JYI) [58] as templates.

## 3. Results

### 3.1. Cyclostome–gnathostome orthology of retinoic acid receptor genes

In both the inshore hagfish *E. burgeri* and the Japanese lamprey *L. japonicum,* three RAR genes, RAR1, RAR2 and RAR3, were previously described, each of which exhibiting tight hagfish–lamprey orthology [20,43]. Furthermore, a fourth lamprey RAR gene, RAR4, was recently identified in *L. japonicum* and two other lamprey species (*L. fluviatilis* and *P. marinus*) [43]. As this lamprey RAR4 gene was shown to be a lamprey-specific duplicate of RAR1 [43], it was not analysed in this study. Previous efforts to determine the relationships between cyclostome and gnathostome RARs employed phylogenetic analyses based on amino acid alignments and produced ambiguous results, albeit tentatively suggesting orthology of RAR1 and RARγ, RAR2 and RARβ, and RAR3 and RARα [20,43].

**Figure 1.**
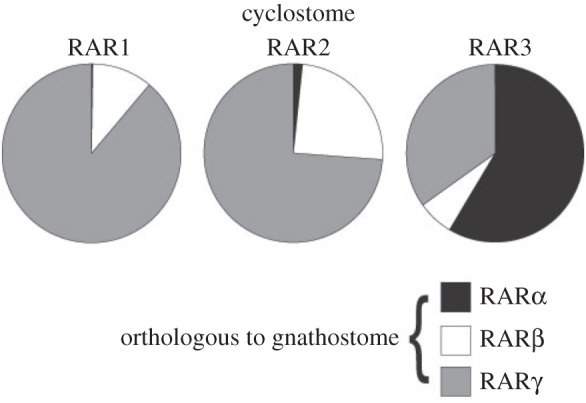
Probabilistic analysis of cyclostome–gnathostome RAR orthologies. Probabilities of orthology of cyclostome RAR subtypes (RAR1, RAR2 and RAR3) to individual gnathostome RAR subtypes (RARα, RARβ and RARγ) are shown as pie charts. The data are based on the maximum-likelihood analyses presented in electronic supplementary material, table S2 and are included as resampling of estimated log-likelihood (RELL) bootstrap probability (BP) values in electronic supplementary material, table S3.

To confirm these relationships as a basis for our comparative study, we performed a probabilistic orthology assessment based on the maximum-likelihood method. We thus computed probabilities of all individual possible tree topologies for five distinct groups (gnathostome RARα, RARβ and RARγ, one of the three cyclostome RAR subtypes, and an outgroup), assuming that the 2R of WGD occurred before the cyclostome–gnathostome split (electronic supplementary material, table S2). Probabilities of tree topologies showing orthology of a given cyclostome to a specific gnathostome RAR gene were added up (electronic supplementary material, table S3). As reported previously, the results of our analysis suggest that RAR1 and RAR3 are likely orthologous to RARγ and RARα, respectively (figure 1). Intriguingly, RAR2 also exhibits a high probability of orthology to RARγ (figure 1), but, of the three cyclostome RAR genes, RAR2 nonetheless is the most likely orthologue of gnathostome RARβ (electronic supplementary material, table S2), a result further supported by probabilistic analyses based on additional tree topology conditions (electronic supplementary material, table S3). Thus, for interpreting the results of the following experiments, we assume an orthology of cyclostome RAR1 to gnathostome RARγ, of cyclostome RAR2 to gnathostome RARβ and of cyclostome RAR3 to gnathostome RARα.

### 3.2. The ligand-binding domain sequences of cyclostome retinoic acid receptors are similar to those of mammalian RARα and RARβ proteins

**Figure 2.**
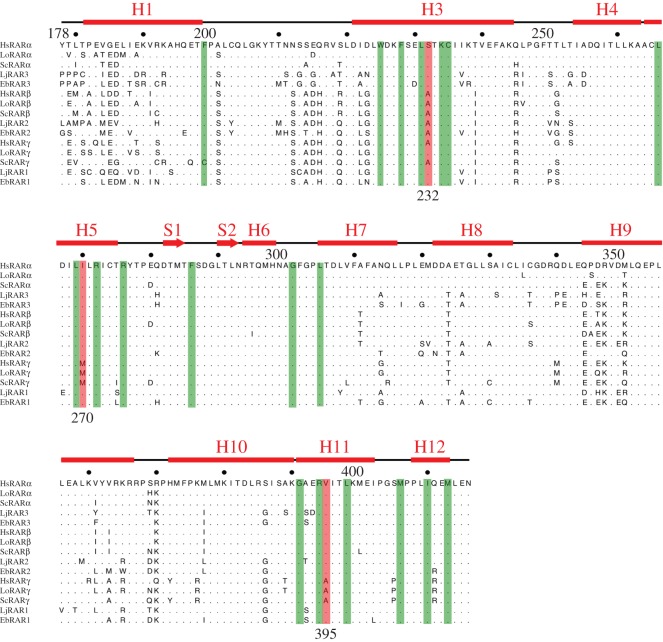
Amino acid conservation within the ligand-binding domains (LBDs) of vertebrate retinoic acid receptor (RAR) proteins. Alignment of the RAR sequences from: human (HsRARα, HsRARβ and HsRARγ), spotted gar (*Lepisosteus oculatus*) (LoRARα, LoRARβ and LoRARγ), small-spotted catshark (*Scyliorhinus canicula*) (ScRARα, ScRARβ and ScRARγ), Japanese lamprey (*Lethenteron japonicum*) (LjRAR1, LjRAR2 and LjRAR3) and inshore hagfish (*Eptatretus burgeri*) (EbRAR1, EbRAR2 and EbRAR3). Amino acids that interact with all-*trans* retinoic acid (ATRA) in human RARs are highlighted in green, and amino acid differences between the three human RAR paralogues RARα, RARβ and RARγ are highlighted in red. The α-helices (H) and β-sheets (S) constituting the RAR LBD are indicated.

The RARs cloned from lamprey and hagfish are highly conserved when compared to the human and mouse RARs. In particular, all amino acids that interact with ATRA in the LBD of the human RARγ [39] are conserved in the cyclostome RARs (figure 2). When considering only the three amino acid positions that differ between the three mammalian RAR paralogues, RARα, RARβ and RARγ, and that were shown to control LBP specificity (i.e. positions 232, 270 and 395) [39], one can observe that both LjRAR1 and EbRAR1 and LjRAR3 and EbRAR3 are characterized by the same amino acid combination (i.e. serine, isoleucine and valine) that is found in mammalian RARα proteins (figure 2). This finding suggests that these four cyclostome receptors have an LBP structure that is similar to that of mammalian RARα. In contrast, for LjRAR2 and EbRAR2, the amino acid combination is the same as that of mammalian RARβ proteins (i.e. alanine, isoleucine and valine; figure 2). The fact that the two cyclostome RAR2 proteins feature the same amino acids as mammalian RARβ proteins supports the orthology between cyclostome RAR2 and gnathostome RARβ. Taken together, these data reveal that of the three paralogous cyclostome RARs, two (RAR2 and RAR3) have amino acid combinations that correspond to their possible human orthologues (RARβ and RARα, respectively), whereas the third one (RAR1) has a RARα-type signature, which contrasts with the results of the probabilistic orthology assessment (figure 1) as well as previous phylogenetic tree analyses [20,43]. Of note, among the six RARs from lamprey and hagfish, none features the amino acid combination seen in the mammalian RARγ. Only the alanine at position 232 of mammalian RARγ is also found in LjRAR2 and EbRAR2, but this amino acid signature is equally present in mammalian RARβ. Given that identical molecular signatures have been described for RAR paralogues in other lamprey species, including *Mordacia mordax*, *P. marinus* and *L. fluviatilis* [20,43], it is likely that these distinctive molecular differences represent a highly conserved feature of cyclostome RARs.

### 3.3. Cyclostome retinoic acid receptors are able to bind paralogue-specific synthetic retinoids and to activate the transcription of target genes upon their binding

**Figure 3.**
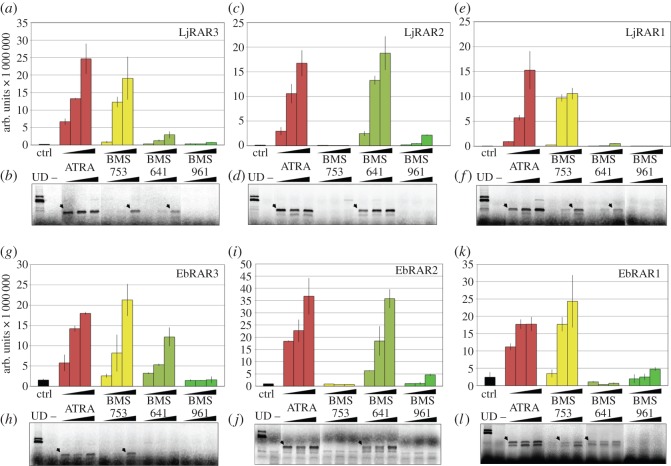
Binding to paralogue-specific synthetic retinoids and transcriptional activity of cyclostome retinoic acid receptors (RARs). (*a*,*c*,*e*) Ability of RARs from the Japanese lamprey *Lethenteron japonicum* (LjRAR3, LjRAR2 and LjRAR1, respectively) to activate the transcription of a luciferase reporter in transfected cells in the presence of increasing concentrations of all-*trans* retinoic acid (ATRA) or of different synthetic retinoids (gnathostome RARα-specific BMS753, gnathostome RARβ-specific BMS641 and gnathostome RARγ-specific BMS961). The ligand-binding domains (LBDs) of the lamprey RARs were fused to a Gal4 DNA binding domain (DBD), and the Gal4 DBD alone was used as a negative control (Ctrl). (*b*,*d*,*f*) Binding of lamprey RARs to ATRA or the different synthetic retinoids at different concentrations was tested by limited proteolysis assays. Protected bands are indicated by arrows and ethanol was used as a negative control (lane -). The undigested protein is also shown (lane UD). (*g*,*i*,*k*) Ability of RARs from the inshore hagfish *Eptatretus burgeri* (EbRAR3, EbRAR2 and EbRAR1, respectively) to activate the transcription of a luciferase reporter in transfected cells in the presence of increasing concentrations of ATRA or of different synthetic retinoids (gnathostome RARα-specific BMS753, gnathostome RARβ-specific BMS641 and gnathostome RARγ-specific BMS961). The LBDs of the hagfish RARs were fused to a Gal4 DBD, and the Gal4 DBD alone was used as a negative control (Ctrl). (*h*,*j*,*l*) Binding of hagfish RARs to ATRA or the different synthetic retinoids at different concentrations was tested by limited proteolysis assays. Protected bands are indicated by arrows and ethanol was used as a negative control (lane -). The undigested protein is also shown (lane UD).

In order to investigate the LBD specificity of the cyclostome RARs, we tested their ability to bind to as well as to be activated by synthetic retinoid compounds that specifically activate the mammalian RAR paralogues RARα, RARβ and RARγ [40–42]. These synthetic retinoids have previously been used to characterize the ligand-binding properties of the single RAR from the invertebrate chordate amphioxus [33]. First, we performed transient transactivation assays with Gal4 DBD–RAR LBD fusion proteins using a luciferase reporter as readout (figure 3). For lamprey LjRAR3, we observed that this receptor was activated by both ATRA and BMS753 (the RARα-selective compound) in a dose-dependent manner (figure 3*a*). We also detected a weak activation with the highest dose of BMS641 (the RARβ-selective compound), but no activation with BMS961 (the RARγ-selective compound). These results suggest that LjRAR3 displays a RARα-type transactivation profile [33,40–42]. Ligand binding assessed by limited proteolysis assays confirmed this conclusion. We observed that in the presence of ATRA, of BMS753 and, to a lesser extent, of BMS641 the receptor was protected from proteolysis (figure 3*b*).

Together, these data indicate that LjRAR3 binds to RARα- and RARβ-selective synthetic retinoids, but is only efficiently activated by BMS753, the RARα-selective compound [33,40–42]. Similar results were obtained with EbRAR3. This receptor was hence activated by ATRA, BMS753 and BMS641 (figure 3*g*), but protected from proteolysis only in the presence of ATRA and BMS753 (figure 3*h*). Only very weak binding was observed with the highest dose of BMS641 (figure 3*h*). Taken together, these data suggest that both cyclostome RAR3 proteins, which phylogenetically show some affinity to gnathostome RARα receptors, display a clear RARα-type ligand-binding specificity [33,40–42]. This suggests that the RAR3s are characterized by an LBP structure that is similar to that of gnathostome RARα receptors [33,40–42].

We next analysed the cyclostome RAR2s. LjRAR2 was activated by ATRA and BMS641 (figure 3*c*) in a dose-dependent manner. In addition, very weak activation was observed with BMS961. These results were confirmed by limited proteolysis assays, in that the receptor was protected from proteolysis in the presence of ATRA and BMS641, but not of BMS961 (figure 3*d*). These results hence suggest that LjRAR2 has a ligand-binding profile that is similar to that of gnathostome RARβ receptors [33,40–42]. An almost identical pattern was observed with EbRAR2: activation by ATRA and BMS641 with a weak effect of the highest dose of BMS961 (figure 3*i*), and a clear binding of ATRA and BMS641, with no detectable signal for BMS961 (figure 3*j*). One can therefore conclude that the cyclostome RAR2s display a ligand specificity and thus very likely a RARβ-type LBP structure [33,40–42], further supporting a possible orthology between the cyclostome RAR2s and the gnathostome RARβs.

Finally, we analysed the cyclostome RAR1s, which are particularly interesting, because, in spite of their phylogenetic association with gnathostome RARγ proteins, their LBP sequence signatures are similar to those of mammalian RARα. LjRAR1 was activated in the presence of ATRA and BMS753 (figure 3*e*). In addition, we detected a very weak activation with high doses of BMS641. Limited proteolysis assays showed that this receptor is able to bind ATRA, BMS753 and, to a lesser extent, BMS641 (figure 3*f*). Of note, neither activation, nor binding was detected with the RARγ-selective compound BMS961 [33,40–42]. For EbRAR1, we observed activation in the presence of ATRA and BMS753, with some activation at the highest dose for BMS961 (figure 3*k*), and a protection from proteolysis in the presence of ATRA, BMS753 and BMS641 (figure 3*l*). Altogether, these results suggest that both LjRAR1 and EbRAR1 have an LBP structure that is largely similar to that of mammalian RARα receptors [33,40–42].

Our data therefore suggest that the structures of the LBPs of LjRAR1, EbRAR1, LjRAR3 and EbRAR3 are similar to those of gnathostome RARα proteins [33,40–42], whereas the LBP structure of LjRAR2 and EbRAR2 is similar to those of gnathostome RARβ receptors [33,40–42]. Interestingly, the RARγ-selective compound BMS961 [33,40–42] stimulated only very weak residual activation in three of the six tested cyclostome RARs (LjRAR2, EbRAR2 and EbRAR1), and none of the six receptors showed any binding to this synthetic retinoid in limited proteolysis assays. From these findings, it can be inferred that RARγ selectivity was acquired specifically in the lineage leading to extant gnathostomes after the split from the cyclostome lineage. Our data also indicate that the pharmacological profile of the cyclostome RAR1s and RAR2s might provide an additional feature for assessing their orthologies with gnathostome RARs.

### 3.4. Structural modelling of the ligand-binding pockets of cyclostome retinoic acid receptors

**Figure 4.**
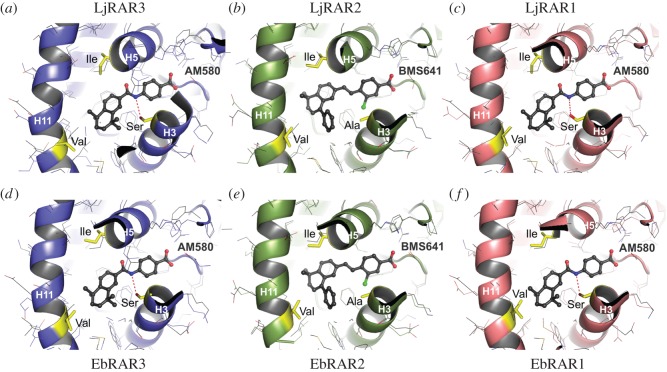
Modelling of the ligand-binding pockets (LBPs) of cyclostome retinoic acid receptors (RARs). Comparison of models of the LBPs of lamprey (*a*–*c*) and hagfish (*d*–*f*) RARs. While RAR1 and RAR3 of both the Japanese lamprey *Lethenteron japonicum* (LjRAR1 and LjRAR3) and the inshore hagfish *Eptatretus burgeri* (EbRAR1 and EbRAR3) were modelled and superimposed on the crystal structure of the ligand-binding domain (LBD) of the human RARα protein bound to the RARα-selective agonist AM580, the RAR2 of both the Japanese lamprey *Lethenteron japonicum* (LjRAR2) and the inshore hagfish *Eptatretus burgeri* (EbRAR2) were modelled and superimposed on the crystal structure of the LBD of the human RARβ protein bound to the RARβ-selective agonist BMS641. Conserved subtype-specific amino acids in α-helices H3, H5 and H11 are highlighted in yellow and labelled. Ligands are shown as ball-and-stick representation. Carbon, oxygen, nitrogen and chlorine atoms are coloured in grey, red, blue and green, respectively. The red dashed lines highlight specific hydrogen bonds between the amide moiety of the human RARα-selective ligand AM580 and the conserved serine residue in the RARα-like cyclostome receptors (LjRAR1 and LjRAR3 as well as EbRAR1 and EbRAR3). Ala, alanine; H, α-helix; Ile, isoleucine; Met, methionine; Ser, serine; Val, valine.

We next used structural modelling approaches to reconstruct the LBDs of the six cyclostome RARs based on the previously characterized X-ray three-dimensional structures of the human RARs. Given the overall sequence conservation within the LBP (figure 2), we used the structures of human RARα and RARβ as templates for our modelling work: while the structure of human RARα in complex with the RARα-selective agonist AM580 (PDB code 1KMR) [57] was used to model the lamprey and hagfish RAR1 and RAR3 proteins, the cyclostome RAR2s were rebuilt with the structure of human RARβ bound to the RARβ-selective ligand BMS641 (PDB code 4JYI) [58]. The resulting cyclostome RAR structure models were subsequently superimposed on the corresponding human RAR structures (i.e. cyclostome RAR1 and RAR3 on human RARα and cyclostome RAR2 on human RARβ; figure 4). The models confirm that the amino acids lining the LBPs of the cyclostome RAR1 and RAR3 proteins are identical to those found in human RARα, including the three subtype-specific residues serine 232 in α-helix H3, isoleucine 270 in α-helix H5 and valine 395 in α-helix H11. In particular, Ser232, which is involved in forming a specific hydrogen bond with human RARα-selective ligands (such as AM580), is conserved in both cyclostome RAR1s and RAR3s (figure 4). Accordingly, as shown in figure 3, these four receptors bind to and are activated by the human RARα-selective agonist BMS753. This contrasts with the situation in the LBP of lamprey and hagfish RAR2, where the serine residue in α-helix H3 is replaced by a smaller alanine. This single substitution allows the cyclostome RAR2 proteins to accommodate the human RARβ-selective agonist BMS641 that contains a bulky chlorine substituent pointing towards alanine 225 (figure 4). This observation is in full agreement with the functional data showing that BMS641 exclusively activates the cyclostome RAR2s (figure 3). In line with our observation that the human RARγ-selective ligand BMS961 fails to bind and activate any of the cyclostome RARs (figure 3), none of the lamprey and hagfish RARs displays residues characteristic of human RARγ, such as a methionine residue in α-helix H5 and a small alanine in α-helix H11 (figure 2). Taken together, our analyses reveal that the LBPs of cyclostome RAR1 and RAR3 selectively accommodate gnathostome RARα-type retinoids, whereas the LBPs of cyclostome RAR2 readily associate with gnathostome RARβ-specific ligands.

### 3.5. Reconstruction of ancestral retinoic acid receptor sequences

**Figure 5.**
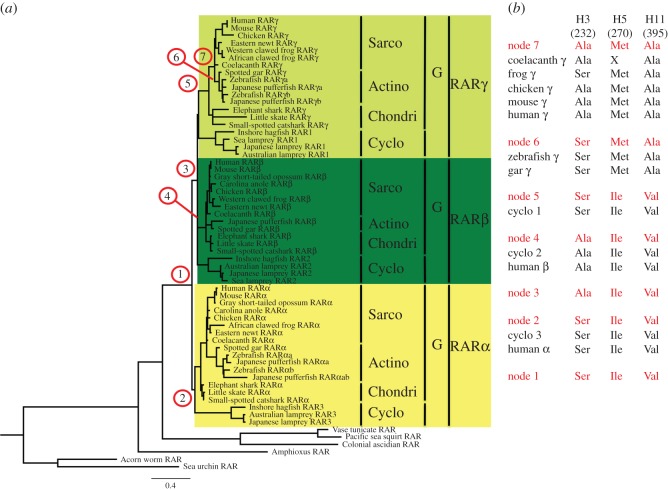
Calculation of ancestral retinoic acid receptor (RAR) sequences of vertebrates. (*a*) Phylogenetic tree of deuterostome RARs. The species represented in the tree are the following: sea urchin (*Strongylocentrotus purpuratus*), acorn worm (*Saccoglossus kowalevskii*), amphioxus (*Branchiostoma floridae*), colonial ascidian (*Polyandrocarpa misakiensis*), vase tunicate (*Ciona intestinalis*), Pacific sea squirt (*Ciona savignyi*), Japanese lamprey (*Lethenteron japonicum*), sea lamprey (*Petromyzon marinus*), Australian lamprey (*Mordacia mordax*), inshore hagfish (*Eptatretus burgeri*), small-spotted catshark (*Scyliorhinus canicula*), little skate (*Leucoraja erinacea*), elephant shark (*Callorhinchus milii*), spotted gar (*Lepisosteus oculatus*), Japanese pufferfish (*Takifugu rubripes*), zebrafish (*Danio rerio*), coelacanth (*Latimeria chalumnae*), Eastern newt (*Notophthalmus viridescens*), Western clawed frog (*Xenopus tropicalis*), African clawed frog (*Xenopus laevis*), Carolina anole (*Anolis carolinensis*), chicken (*Gallus gallus*), grey short-tailed opossum (*Monodelphis domestica*), house mouse (*Mus musculus*) and human (*Homo sapiens*). The extracted ancestral sequences are: node 1, prior to the diversification of RARα, RARβ, and RARγ; node 2, at the base of the RARα group; node 3, prior to the divergence of RARβ and RARγ; node 4, at the base of the RARβ group; node 5, at the base of the RARγ group; node 6, at the base of the actinopterygian RARγ group; node 7, at the base of the sarcopterygian RARγ group. Note that some incomplete sequences have not been included in the analysis. (*b*) RAR amino acid residues located at the key positions of the ligand-binding pocket (LBP) in α-helix H3 (amino acid position 232), α-helix H5 (amino acid position 270) and α-helix H11 (amino acid position 395). Actino, actinopterygians; Ala, alanine; Chondri, chondrichthyans; Cyclo, cyclostomes; G, gnathostomes; H, α-helix; Ile, isoleucine; Met, methionine; Sarco, sarcopterygians; Ser, serine; Val, valine; X, not known.

The above results suggest a discrepancy between the phylogenetic history of some RAR sequences and their functional characteristics. In particular, our data suggest that RARγ proteins acquired their specific LBP architecture after the cyclostome–gnathostome split. Therefore, to gain insights into the evolutionary history of amino acid substitutions in vertebrate RARs, we generated ancestral sequences of RAR LBDs at different evolutionary divergence nodes in the chordate tree of life (figure 5). Using the phylogenetic association of the three cyclostome RARs (RAR3, RAR2 and RAR1) with one of the three gnathostome RAR subtypes (RARα, RARβ and RARγ, respectively), the ancestral sequences were extracted at the node prior to the vertebrate-specific two rounds of WGDs and thus the origin of RARα, RARβ and RARγ (node 1), at the base of RARα (node 2), at the node prior to the divergence of RARβ and RARγ (node 3), RARβ (node 4) and RARγ (node 5) as well as at the base of the actinopterygians (node 6) and sarcopterygians (node 7) for RARγ. For each node, the best predicted sequence was retained. In the resulting ancestral sequences, some positions could be predicted with high accuracy and statistical support, whereas other positions were more ambiguous, as highlighted by the sequence visualization method used to compile electronic supplementary material, figure S3.

From these reconstructions, we were able to deduce that the ancestral RAR, prior to duplication and divergence into RARα, RARβ and RARγ (node 1) was characterized by a RARα-like arrangement of the three key amino acids in α-helices H3, H5 and H11 (serine, isoleucine and valine, respectively). Consistently, the same observation can be made for the receptors at the base of RARα (node 2), and, strikingly, also for the receptors at the base of RARγ (node 5). In contrast, the receptor present at the base of RARβ and RARγ (node 3) was characterized by a RARβ-like signature (alanine, isoleucine and valine), which was coherently also recovered for the base of RARβ (node 4). These observations suggest that the single, unduplicated ancestral RAR at the base of the vertebrate lineage was RARα-like and that a duplicated vertebrate paralogue acquired a RARβ-like LBP structure before the second WGD by mutation of a serine into an alanine in α-helix H3. Our calculations further indicate a number of significant additional changes in the course of RARγ diversification, which thus occurred after the second WGD. Thus, while cyclostome RAR1 group members have reverted to an ancestral RARα-like pattern (serine, isoleucine and valine), gnathostome RARγ receptors have acquired a methionine in H5 and an alanine in H11 (node 7). Furthermore, in both actinopterygian (node 6) and frog RARγ proteins the alanine in H3 has been reverted to the ancestral serine. Of note, the poor quality of the sequence did not allow an unequivocal identification of amino acid 270 in the H5 of coelacanth RARγ. As the genome sequences of additional deuterostome species become available, an improved taxon sampling is likely to further improve the resolution of ancestral sequence reconstruction analyses targeting RARs [33].

## 4. Discussion

Our analyses of the cyclostome RARs confirm and extend our previous study, which was mainly focusing on invertebrate chordate RARs [33]. The results suggest that paralogue specificity of the LBPs of gnathostome RARs has emerged earlier for RARα and RARβ than for RARγ. For RARγ, we found evidence for a stepwise process, in which mutations in key residues accumulated over time, with the typical RARγ arrangement (alanine, methionine, alanine) found in mammals and birds having been established by an initial mutation in α-helix H3, which was followed by mutations in α-helix H5 and α-helix H11.

### 4.1. The cyclostome retinoic acid receptor complement

We have characterized the LBP structure of six RARs from two cyclostomes, the lamprey *L. japonicum* and the hagfish *E. burgeri*. Each one of these RARs is able to bind ATRA and activate the transcription of target gene constructs in the presence of this endogenous retinoid. Based on molecular phylogenetic analyses in this and previous studies [20,43], it is most likely that each of the three cyclostome RAR genes (RAR1, RAR2 and RAR3) show a one-to-one orthology to one of the three gnathostome RAR genes (RARγ, RARβ and RARα, respectively). In addition, as mentioned above, a fourth RAR has been identified in lampreys (RAR4), which appears to be a lineage-specific duplicate of lamprey RAR1 [43]. These hypothesized orthology relationships between the cyclostome and gnathostome RAR genes are in accordance with the pharmacological profiles we obtained for RAR2 and RAR3. In contrast, the differences in the pharmacological signatures of the RAR1s and the RARγ proteins are of major importance, because RAR1 proteins do not display the typical mammalian RARγ LBP sequence signature, although the association of RAR1 and RARγ is the most strongly supported grouping in phylogenetic analyses.

### 4.2. Evolution of the ligand-binding pocket of cyclostome retinoic acid receptors

Our results suggest that the cyclostome RARs acquired their differential ligand specificity by mutation and reversion of a single LBP amino acid at position 232 of α-helix H3, although we cannot rule out the possibility that amino acid changes outside the LBP have also contributed to this process [59]. Given that paralogous lamprey and hagfish receptors exhibit very similar, if not identical, ligand specificity profiles, which are likely the result of the conservation of key amino acids associated with the LBP, we expect that the conclusions drawn from this study can be generalized and applied to all cyclostomes.

On a more technical level, on some occasions, we observed slight differences between the transactivation and the ligand-binding results. This is, for example, the case for the RARβ-selective compound BMS641, for which there was a clear binding to the *L. japonicum* RAR1 and RAR3, but only a weak transactivation. A similar situation was previously observed with zebrafish RARα and, more generally, with mammalian RARs, as it was shown that all mammalian RARs can bind BMS641, but only RARβ could be activated in its presence [33,41]. We also found that BMS641 was able to bind to *E. burgeri* RAR1, but, in this case, there was no significant activation of the receptor, suggesting that BMS641 might exert an antagonist effect on the transactivation potential of this receptor, hence exhibiting an inverted dose–response relationship. A similar phenomenon using this synthetic retinoid has previously been observed for other RARs (notably for the RARγ of the frog *Xenopus laevis*) [33]. Finally, there was one case, *E. burgeri* RAR3 in association with BMS641, in which we observed activation of the receptor in the presence of the synthetic retinoid, but no clear binding. This result might be due to the sensitivity of the limited proteolysis assay, which might not have allowed us to reveal the physical binding of *E. burgeri* RAR3 to BMS641 [41]. However, taken everything together, the discrepancies between the two methods are minor and do not significantly affect the main conclusions: while cyclostome RAR3 and RAR2 have a ligand specificity that corresponds to their phylogenetic association with gnathostome RARs, this is not the case for cyclostome RAR1.

For vertebrate RARs in general, the differences between RARα/RARβ and RARγ may reflect differences of evolutionary constraints acting on the RAR LBP. Thus, although ATRA is the main active retinoid and although biological functions of oxidized ATRA metabolites have been dismissed on genetic grounds in mice [38], one cannot exclude that some differences in ligand-binding specificity might have functional implications and have thus been fixed and conserved in specific RAR paralogues. Indeed, there is accumulating evidence that alternative ligands do exist for several NRs. For example, it has been shown that the testosterone metabolite 5α-androstane-3β,17β-diol competes with 17β-oestradiol for binding to the oestrogen receptor ERβ in the prostate, where this alternative ERβ ligand has been shown to regulate cell proliferation and hence organ growth [60]. Furthermore, another alternative steroid, 27-hydroxycholesterol, has recently been characterized and has allowed the linking of hypercholesterolemia to breast cancer development [61]. Similarly, although 1α,25-dihydroxyvitamin D3 is generally regarded as the natural ligand of the vitamin D receptor VDR, in the intestine this receptor is apparently activated by lithocholic acid, a bile acid [62]. These examples show that NRs, for which the main endogenous ligands have already been described, can, under specific conditions, associate with other molecules and trigger tissue-specific effects with significant biological and thus functional impact. For RARs, some other retinoids, such as 9-*cis*- or 13-*cis*-retinoic acid, might act as alternative ligands and this intriguing pharmacological problem will certainly be investigated in the future [63,64]. In this context, it can already be hypothesized that the constraints on the LBP are not going to be identical for RARγ and RARα/RARβ, and it will thus be important to determine if these constraint differences are linked to a relaxation of evolutionary pressure or to a positive selection for a specific function.

### 4.3. An evolutionary scenario for retinoic acid receptor ligand-binding pocket evolution

**Figure 6.**
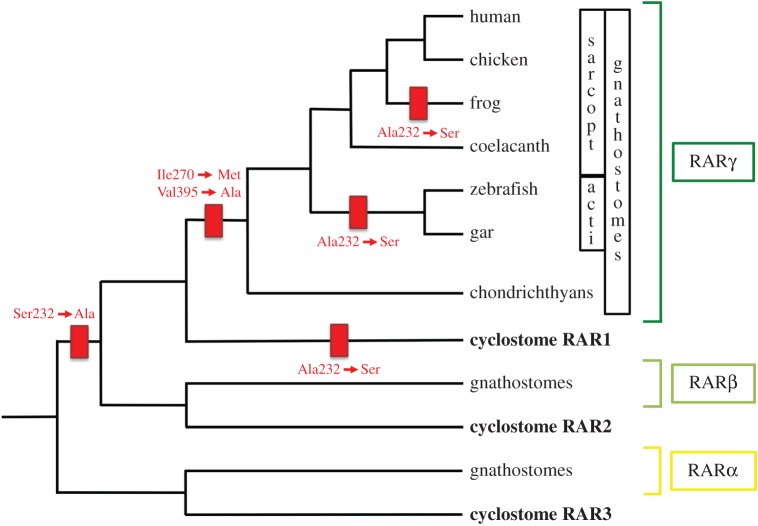
Inferred evolutionary history of retinoic acid receptors (RARs) and their ligand-binding pocket (LBP) structure in vertebrates. A simplified phylogeny of vertebrate RARs is shown. Indicated in red are mutation events in the RAR LBP that occurred in the course of vertebrate evolution. The ancestral vertebrate RAR was characterized by an LBP with a serine at position 232 of α-helix H3, an isoleucine at position 270 of α-helix H5, and a valine at position 395 of α-helix H11. In extant vertebrates, this signature can be found in both cyclostome RAR3 and gnathostome RARα receptors. Very early during vertebrate diversification, before the cyclostome–gnathostome split, a serine to alanine mutation took place at position 232 of α-helix H3 in a receptor ancestral to both the RAR2/RARβ and RAR1/RARγ groups. This mutation was subsequently reverted in an ancestral cyclostome RAR1 receptor. Within the gnathostome RARγ group, two mutations occurred before the diversification of the chondrichthyans: isoleucine to methionine at position 270 of α-helix H5 and valine to alanine at position 395 of α-helix H11. Furthermore, reversals of the mutation at position 232 of α-helix H3 occurred in ancestral RARγ receptors of both actinopterygians and frogs. Acti, actinopterygians; Ala, alanine; Ile, isoleucine; Met, methionine; sarcopt, sarcopterygians; Ser, serine; Val, valine.

Altogether, our results allow the development of a scenario for RAR evolution and diversification of LBP structure following the WGDs in vertebrates (figure 6). We propose that the structure of the LBP of the ancestral vertebrate RAR diverged rapidly after the WGDs, i.e. before the cyclostome–gnathostome split, by specific amino acid mutations (e.g. in an ancestral RARβ/RARγ receptor, by mutation of a serine to an alanine at position 232 in H3). The pocket structure then remained largely unchanged in the gnathostome RARα and RARβ lineages, which might be due to specific selective constraints that remain to be determined. This hypothesis is coherent with results obtained from the single RAR of the invertebrate chordate amphioxus [33], which diverged before the vertebrate-specific 2R WGD and represents the closest available proxy for the ancestral chordate RAR (figure 5). Amphioxus RAR is characterized by a cysteine at position 232 in H3, which does not correspond to any of the three mammalian RAR paralogues, as well as by an isoleucine at position 270 and a valine at position 395, both of which are conserved with both mammalian RARα and RARβ [33]. The amphioxus RAR is further able to bind both RARα- and RARβ-selective compounds (i.e. BMS753 and BMS641, respectively), but not the RARγ-specific BMS961, and activates transcription of a reporter gene exclusively in the presence of the RARβ-selective retinoid BMS641 [33].

In the gnathostome RARγ lineage, the LBP structure changed, again by accumulation of specific amino acid mutations. From the ancestral sequence reconstruction analysis, one can infer that, following the mutation of a serine to an alanine at position 232 in H3 of an ancestral RARβ/RARγ, H5 (isoleucine to methionine at position 270) and H11 (valine to alanine at position 395) were mutated in an ancestral gnathostome RARγ receptor. Of note, while the mutation in H3 of an ancestral RARβ/RARγ likely occurred before the second round of WGD, the mutations in H5 and H11 probably took place in an ancestral RARγ after the second round of WGD. In both the actinopterygian and frog RARγ lineages, the amino acid at position 232 in H3 was subsequently submitted to independent mutation events leading to the reversion of the alanine to a serine. The same reversion also took place independently in cyclostome RAR1 receptors. It is thus likely that this LBP position in RARγ receptors is not subjected to strong evolutionary constraints. The complex evolutionary history of RARγ contrasts with that of RARβ, where the mutation from a serine to an alanine at position 232 occurred very early during vertebrate evolution, in an ancestral RARβ/RARγ receptor following the first round of WGD, and was subsequently fixed and thus remained unchanged in the course of RARβ evolution. When compared with the ancestral, RARα-like state, the mutations and reversions we observed at LBP position 232 in RARβ/RARγ receptors (figures 2 and5) are good examples of convergent mutations in duplicated genes (i.e. paralogues) that experience independent evolutionary fates following duplication. This situation is reminiscent of convergent mutations that we have previously described for RARs at the serine at position 369 in the loop between H9 and H10, which is an important phosphorylation site controlling the activity of the receptor [65]. This residue was mutated to a serine independently at least four times in RAR evolution and, just like LBP position 232, exhibits a differential mutational history in different RAR paralogues.

This study, together with our previous analyses [33,65], converge on the notion that the functional characterization of NRs from non-model organisms, combined with in-depth phylogenetic analyses, can shed light both on evolutionary processes and on the plasticity of NRs, which are important pharmaceutical targets [66]. These comparative approaches thus represent a very valuable complement to the intricate studies of NRs in classic model organisms and provide valuable insights into the molecular tinkering that occurred in the course of evolution and that actively shaped the diversity of protein form and functions observed today.

## Supplementary Material

Supplementary Figure S1. Amino acid residues selected for probabilistic orthology assessment of retinoic acid receptor (RAR) sequences.

## Supplementary Material

Supplementary Figure S2. Alignment of the ligand-binding domains (LBDs) of 61 retinoic acid receptor (RAR) sequences retained for ancestral sequence reconstruction.

## Supplementary Material

Supplementary Figure S3. Ancestral retinoic acid receptor (RAR) sequences calculated at seven nodes of the chordate tree of life.

## Supplementary Material

Supplementary Table S1. Accession numbers of the retinoic acid receptor (RAR) sequences used for probabilistic orthology assessment.

## Supplementary Material

Supplementary Table S2. Probabilistic assessment of orthologies of cyclostome retinoic acid receptor (RAR) genes based on the maximum likelihood (ML) method.

## Supplementary Material

Supplementary Table S3. Sums of probabilities of individual tree topologies for cyclostome-gnathostome retinoic acid receptor (RAR) orthologies.

## Supplementary Material

Supplementary Table S4. Accession numbers of the retinoic acid receptor (RAR) sequences used for ancestral sequence reconstruction.

## Data accessibility

Supporting data for this article are included in the electronic supplementary material. Additional raw data are available from the authors upon request.
