# Correction to ‘Evolutionary diversification of retinoic acid receptor ligand-binding pocket structure by molecular tinkering'

**DOI:** 10.1098/rsos.160895

**Published:** 2016-12-14

**Authors:** Julianan Gutierrez-Mazariegos, Eswar Kumar Nadendla, Romain A. Studer, Susana Alvarez, Angel R. de Lera, Shigehiro Kuraku, William Bourguet, Michael Schubert, Vincent Laudet

*R. Soc. open. sci*. **3**, 150484. (16 March 2016). (doi:10.1098/rsos.150484)

Figure 2 was presented incorrectly in the paper. The corrected figure is presented below:

**Figure 2.**
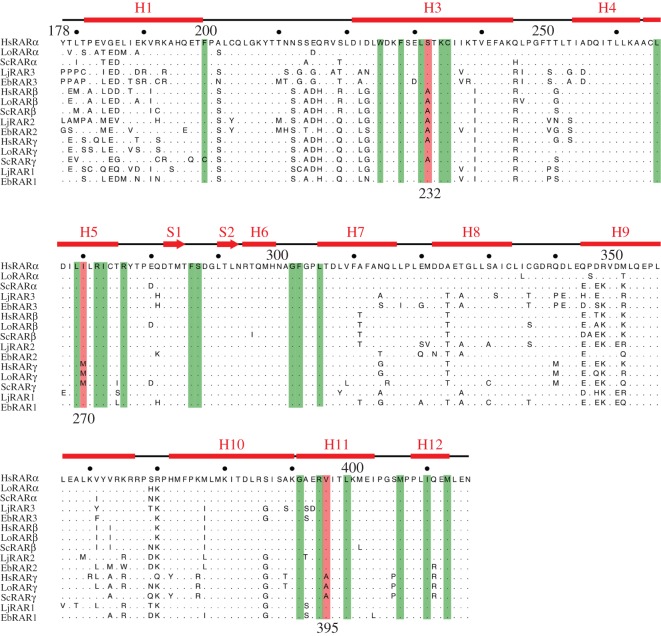
Amino acid conservation within the ligand-binding domains (LBDs) of vertebrate retinoic acid receptor (RAR) proteins. Alignment of the RAR sequences from: human (HsRARα, HsRARβ and HsRARγ), spotted gar (*Lepisosteus oculatus*) (LoRARα, LoRARβ and LoRARγ), small-spotted catshark (*Scyliorhinus canicula*) (ScRARα, ScRARβ and ScRARγ), Japanese lamprey (*Lethenteron japonicum*) (LjRAR1, LjRAR2 and LjRAR3) and inshore hagfish (*Eptatretus burgeri*) (EbRAR1, EbRAR2 and EbRAR3). Amino acids that interact with all-*trans* retinoic acid (ATRA) in human RARs are highlighted in green, and amino acid differences between the three human RAR paralogues RARα, RARβ and RARγ are highlighted in red. The α-helices (H) and β-sheets (S) constituting the RAR LBD are indicated.

