# Supplementary material for: Evolutionary diversification of retinoic acid receptor ligand-binding pocket structure by molecular tinkering
Source: R Soc Open Sci. 2016 Mar 16;3(3):150484. doi: 10.1098/rsos.150484 (PMC4821253; doi:10.1098/rsos.150484)
Supplement: Supplementary Figure S1. Amino acid residues selected for probabilistic orthology assessment of retinoic acid receptor (RAR) sequences. [file rsos150484supp1.pdf]

Group	Species	1–153
Gnathostome RARα	human	QDKSSGYHYGVSACEGCKGFFRRSIQKNMVYTCHRDKNCIINKVTRNRCQYCRLQKCFEVGMSKESVRNDRNESYTLTPEVGELIEKVRKAHQETFPALCQLGKYTTNSLDIDLWDKFSELSTKCIIKTVEFAKQLPGFTTLTIADQITLLKA
	chicken	QDKSSGYHYGVSACEGCKGFFRRSIQKNMVYTCHRDKNCIINKVTRNRCQYCRLQKCFEVGMSKESVRNDRNESYIVTPEVEELIEKVRKAHQETFPALCQLGKYTTNSLDIDLWDKFSELSTKCIIKTVEFAKQLPGFTTLTIADQITLLKA
	Western clawed frog	QDKSSGYHYGVSACEGCKGFFRRSIQKNMVYTCHRDKNCIINKVTRNRCQYCRLQKCFEVGMSKESVRNDRNESYVLSPETEDLIEKVRKAHQETFPALCQLGKYTTNSLDIDLWDKFSELSTKCIIKTVEFAKQLPGFTTLTIADQITLLKS
	spotted gar	QDKSSGYHYGVSACEGCKGFFRRSIQKNMVYTCHREKNCIINKVTRNRCQYCRLQKCLEVGMSKESVRNDRNESYVLSPATEDMIAKVRKAHQETFPSLCQLGKYTTNSLDIDLWDKFSELSTKCIIKTVEFAKQLPGFTTLTIADQITLLKA
	elephant shark	QDKSSGYHYGVSACEGCKGFFRRSIQKNMVYTCHRDKNCIINKVTRNRCQYCRLQRCLEVGMSKESVRNDRNESYTITPEIEDLIEKVRKAHQETFPALCQLGKYTTNTLDIDLWDKFSELSTKCIIKTVEFAKHLPGFTTLTIADQITLLKA
	small-spotted catshark	QDKSSGYHYGVSACEGCKGFFRRSIQKNMVYTCHRDKNCIINKVTRNRCQYCRLQKCLEVGMSKESVRNDRNESYTITPETEDLIEKVRKAHQETFPALCQLGKYTTNTLDIDLWDKFSELSTKCIIKTVEFAKHLPGFTTLTIADQITLLKA
Gnathostome RARβ	human	QDKSSGYHYGVSACEGCKGFFRRSIQKNMIYTCHRDKNCVINKVTRNRCQYCRLQKCFEVGMSKESVRNDRNESYEMTAELDDLTEKIRKAHQETFPSLCQLGKYTTNRLDLGLWDKFSELATKCIIKIVEFAKRLPGFTGLTIADQITLLKA
	chicken	QDKSSGYHYGVSACEGCKGFFRRSIQKNMVYTCHRDKNCVINKVTRNRCQYCRLQKCFEVGMSKESVRNDRNENYEMTAELDDLTEKIRKAHQETFPSLCQLGKYTTNRLDLGLWDKFSELATKCIIKIVEFAKRLPGFTSLTIADQITLLKA
	Western clawed frog	QDKSSGYHYGVSACEGCKGFFRRSIQKNMVYTCHREKNCVINKVTRNRCQYCRLQRCFEVGMSKESVRNDRNENYEMTAELDDLTEKIRKAHQETFPSLCQLGKYTTNRLDLGLWDKFSELATKCIIKIVEFAKRLPGFTSLTIADQITLLKA
	spotted gar	QDKSSGYHYGVSACEGCKGFFRRSIQKNMVYTCHREKNCVINKVTRNRCQYCRLQRCFEVGMSKESVRNDRNESYELTAELEDLIAKIRKAHQETFPSLCQLGKYTTNRLDLGLWDKFSELATKCIIKIVEFAKRVPGFTGLTIADQITLLKA
	elephant shark	QDKSSGYHYGVSACEGCKGFFRRSIQKNMVYTCHREKNCVINKVTRNRCQYCRLQKCFEVGMSKESVRNDRNESYTMTAELEDLIEKICKAHQETFPSLCQLGKYTTNRLDLGLWDKFSELATKCIIKIVEFAKRLPGFTSLTIADQITLLKA
	small-spotted catshark	QDKSSGYHYGVSACEGCKGFFRRSIQKNMVYTCHREKNCVINKVTRNRCQYCRLQKCFEVGMSKESVRNDRNESYTMTAELEDLIEKICKAHQETFPSLCQLGKYTTNRLDLGLWDKFSELATKCIIKIVEFAKRLPGFTSLTIADQITLLKA
Gnathostome RARγ	human	NDKSSGYHYGVSSCEGCKGFFRRSIQKNMVYTCHRDKNCIINKVTRNRCQYCRLQKCFEVGMSKEAVRNDRNDSYELSPQLEELITKVSKAHQETFPSLCQLGKYTTNQLDLGLWDKFSELATKCIIKIVEFAKRLPGFTGLSIADQITLLKA
	chicken	NDKSSGYHYGVSSCEGCKGFFRRSIQKNMVYTCHRDKNCQINKVTRNRCQYCRLQKCFEVGMSKEAVRNDRNDSYEMTPELEELIQKVSKAHQETFPSLCQLGKYTTNQLDLGLWDKFSELATKCIIKIVEFAKRLPGFTTLSIADQITLLKA
	Western clawed frog	NDKSSGYHYGVSSCEGCKGFFRRSIQKNMVYTCHRDKNCQINKVTRNRCQFCRLQKCFQVGMSKEAVRNDRNDSYEMPPEMEELIQKVSKAHQETFPSLCQLGKYTTNQLDLGLWDKFSELSTKCIIKIVEFAKRLPGFTTLTIADQITLLKS
	spotted gar	QDKSSGYHYGVSSCEGCKGFFRRSIQKNMVYTCHRDKNCQINKVTRNRCQYCRLQKCFEVGMSKEAVRNDRNESYELSSELEELVSKVSKAHQETFPSLCQLGKYTTNQLDLGLWDKFSELSTKCIIKIVEFAKRLPGFTTLTIADQITLLKA
	elephant shark	QDKSSGYHYGVSSCEGCKGFFRRSIQKNMVYTCHRDKSCTINKVTRNRCQYCRLHKCFQVGMSKESVRNDRNESLPVAAETEDLIQKVCRAHQETFPSLCQLGKYTTNQLDLGLWDKFSELSTKCIIKIVEFAKRLPGFTGLTIADQITLLKA
Cyclostome RAR1	inshore hagfish	QDKSSGYHYGVSSCEGCKGFFRRSVQKNMVYTCHRDKNCVINKNTRNRCQYCRLQKCFEVGMSKEAVRNDRNERYELSCEQEQLVDKIRKSHQETFPSLCQLGKYTTNQLDLGLWDKFSELSTKCIVKIVEFAKRLPGFPSLTIADQITLLKA
	Japanese lamprey	QDKSSGYHYGVSSCEGCKGFFRRSIQKNMVYTCHRDKNCVINKNTRNRCQYCRLQRCFQVGMSKEAVRNDRNECYTLSPELEDMINKINKAHQETFPSLCQLGKYTTNQLDLNLWDKFSELSTKCIVKIVEFAKRLPGFPSLTIADQITLLKA
Cyclostome RAR2	inshore hagfish	QDKSSGYHYGVSSCEGCKGFFRRSIQKNMVYTCPRDKGCVINKVTRNRCQYCRLQKCFEVGMSKESVRNDRNETLAMPAEMEVLIEKVHKAHQETFPSLYQLGKYTMNQLDLGLWDKFSELATKCIIKIVEFAKRLPGFVNLSIADQITLLKA
	Japanese lamprey	QDKSSGYHYGVSSCEGCKGFFRRSIQKNMSYTCHREKCCVINKVTRNRCQYCRLQKCFEVGMSKESVRNDRNENGSLTPEMEELVEKVRKAHEETFPSLYQLGKYTMHQLDLSLWDKFSELATKCIVKIVEFAKHLPGFVGLSIADQITLLKA
Cyclostome RAR3	inshore hagfish	QDKSSGYHYGVSSCEGCKGFFRRSIQKNMVYTCHRDKKCIINKITRNRCQYCRLQRCLEVGMSKESVRNDRSEIPPPCPEIEDLIDRVRRAHQETFPSLCQLGKYTTSTLDANLWDKFSELSTKCIVKTVEFAKRIPGFSTLGIDDQITLLKA
	Japanese lamprey	QDKSSGYHYGVSSCEGCKGFFRRSIQKNMVYTCHRDKKCVVNKITRNRCQYCRLQRCLEVGMSKESVRNDRSELPPAPPELEDLTSRVCRAHQETFPNLCQLGKYTMTTLDADLWDKFSDLSTKCIVRTVEFAKRIPGFSTLAIDDQITLLKA
Outgroup	*Ciona intestinalis*	GDKSSGYHYGVASCEGCKGFFRRSVQKNMQYTCHRNKQCLINKSTRSRCQYCRLQKCIQAGMLRESVRNDRNDEPSCSPEIEALVTSVHKFHVETFPLSSELKKYQIPKTDSNLWEKFAELSTKCIVKIVEFAKGIPGFQDFTIADQITLLKC
	Ciona savignyi	GDKSSGYHYGVASCEGCKGFFRRSVQKNMQYTCHRNKQCLINKSTRSRCQFCRLQKCFQAGMLRESVRNDRNDDVTCSPEIEALVASVYKYHVDTFPLNSNLRKYKIAKTDSNLWEKFAELSTKCIVKIVEFAKGVPGFQDFTIADQITLLKC
	Polyandrocarpa misakiens	GDKSSGYHYGVASCEGCKGFFRRSVQKNMQYTCHRNKNCVINKSTRSRCQYCRLQKCFQVGMLKESVRNDRNEEIVVTPEIENIVSVVAKAHLDTFPKNEDLNKYNTAPVDVKLWSKFSDLSTKSIIKIVEFAKSVPGFTDLTIADQITLLKS
	Branchiostoma floridae	SDKSSGYHYGVASCEGCKGFFRRSIQKNMQYVCHRDKNCVINKVTRNRCQFCRLKKCFDVGMSKESVRNDRNLSYNWTPEIQTIITTVREAHMATLPDMGKLPKYKVKPTDIELWQHFSDLCTETIIKIVQFAKKVPGFTTFGTADQITLLKA

Group	Species	154–306
Gnathostome RARα	human	ACLDILILRICTRYTPEQDTMTFSDGLTLNRTQMHNAGFGPLTDLVFAFANQLLPLEMDDAETGLLSAICLICGDRQDLEQPDRVDMLQEPLLEALKVYVRKRRPSRPHMFPKMLMKITDLRSISAKGAERVITLKMEIPGSMPPLIQEMLEN
	chicken	ACLDILILRICTRYTPEQDTMTFSDGLTLNRTQMHNAGFGPLTDLVFAFANQLLPLEMDDAETGLLSAICLICGDRQDLEQPDKVDKLQEPLLEALKIYVRKRRPNKPHMFPKMLMKITDLRSISAKGAERVITLKMEIPGSMPPLIQEMLEN
	Western clawed frog	ACLDILILRICTRYTPDQDTMTFSDGLTLNRTQMHNAGFGPLTDLVFAFANQLLPLEMDDAETGLLSAICLICGDRQDLEQPDKVDKLQEPLLEALKIYVRNRRPKKPHMFPKMLMKITDLRSISAKGAERVITLKMEIPGSMPPLIQEMLEN
	spotted gar	ACLDILILRICTRYTPEQDTMTFSDGLTLNRTQMHNAGFGPLTDLVFAFANQLLPLEMDDAETGLLSAICLLCGDRQDLEQSDRVDTLQEPLLEALKVYVRKRRPHKPHMFPKMLMKITDLRSISAKGAERVITLKMEIPGSMPPLIQEMLEN
	elephant shark	ACLDILILRICTRYTPDQDTMTFSDGLTLNRTQMHNAGFGPLTDLVFAFANQLLPLEMDDAETGLLSAICLICGDRQDLEQAEKVDKLQEPLLEALKIYVRKRRPNKPHMFPKMLMKITDLRSISAKGAERVITLKMEIPGSMPPLIQEMLEN
	small-spotted catshark	ACLDILILRICTRYTPDQDTMTFSDGLTLNRTQMHNAGFGPLTDLVFAFANQLLPLEMDDAETGLLSAICLICGDRQDLEEPEKVDKLQEPLLEALKIYVRKRRPNKPHMFPKMLMKITDLRSISAKGAERVITLKMEIPGSMPPLIQEMLEN
Gnathostome RARβ	human	ACLDILILRICTRYTPEQDTMTFSDGLTLNRTQMHNAGFGPLTDLVFTFANQLLPLEMDDTETGLLSAICLICGDRQDLEEPTKVDKLQEPLLEALKIYIRKRRPSKPHMFPKILMKITDLRSISAKGAERVITLKMEIPGSMPPLIQEMLEN
	chicken	ACLDILILRICTRYTPEQDTMTFSDGLTLNRTQMHNAGFGPLTDLVFTFANQLLPLEMDDTETGLLSAICLICGDRQDLEEPMKVDKLQEPLLEALKIYIRKRRPNKPHMFPKILMKITDLRSISAKGAERVITLKMEIPGSMPPLIQEMLEN
	Western clawed frog	ACLDILILRICTRYTPEQDTMTFSDGLTLNRTQMHNAGFGPLTDLVFTFANQLLPLEMDDTETGLLSAICLICEDRQDLEEPAKVDKLQEPLLEALKIYIRKRRPNKPHMFPKILMKITDLRSISAKGAERVITLKLEIPGSMPPLIQEMLEN
	spotted gar	ACLDILILRICTRYTPDQDTMTFSDGLTLNRTQMHNAGFGPLTDLVFTFANQLLPLEMDDTETGLLSAICLISGDRQDLEEPAKVDKLQEPLLEALKIYIRKRRPSKPHMFPKILMKITDLRSISAKGAERVITLKMEIPGSMPPLIQEMLEN
	elephant shark	ACLDILILRICTRYTPDQDTMTFSDGLTLNRTQMHNAGFGPLTDLVFTFANQLLPLEMDDTETGLLSAICLICGDRQDLEDAGKVDKLQEPLLEALKIYIRKRRPNKPHMFPKILMKITDLRSISAKGAERVITLKLEIPGSMPPLIQEMLEN
	small-spotted catshark	ACLDILILRICTRYTPEQDTMTFSDGLTLNRIQMHNAGFGPLTDLVFTFANQLLPLEMDDTETGLLSAICLICGDRQDLEDAEKVDKLQEPLLEALKIYIRKRRPNKPHMFPKILMKITDLRSISAKGAERVITLKLEIPGSMPPLIQEMLEN
Gnathostome RARγ	human	ACLDILMLRICTRYTPEQDTMTFSDGLTLNRTQMHNAGFGPLTDLVFAFAGQLLPLEMDDTETGLLSAICLICGDRMDLEEPEKVDKLQEPLLEALRLYARRRRPSQPYMFPRMLMKITDLRGISTKGAERAITLKMEIPGPMPPLIREMLEN
	chicken	ACLDILMLRICTRYTPEQDTMTFSDGLTLNRTQMHNAGFGPLTDLVFAFAGQLLPLEMDDTETGLLSAICLICGDRMDLEEPEKVDKLQEPLLEALKIYARRRRPSKPYMFPRMLMKITDLRGISTKGAERAITLKMEIPGPMPPLIREMLEN
	Western clawed frog	ACLDILMLRICTRYTPEQDTMTFSDGLTLNRTQMHNAGFGPLTDLVFAFADQLLPLEMDDTETGLLSAICLICGDRMDLEEPEKVEKLQEPLLEALKFYARRRRPDKPYMFPRMLMKITDLRGISTKGAERAITLKMEIPGPMPPLIREMLEN
	spotted gar	ACLDILMLRICTRYTPEQDTMTFSDGLTLNRTQMHNAGFGPLTDLVFAFAGQLLPLEMDDTETGLLSAICLICGDRMDLEEPQKVDRLQEPLLEALKLYARRRRPNKPHMFPRMLMKITDLRGISTKGAERAITLKMEIPGPMPPLIREMLEN
	elephant shark	ACLDILMLRICTRYTPDQDTMTFSDGLTLNRTQMHNAGFGPLTDLVFAFADRLLPLEMDDTETGLLSAICLICGDRMDLEEPEKVDQLQEPLLEALKLYARRRRPDKPYMFPRMLMKITDLRGISAKGAERAITLKMEIPGPMPPLIREMLEN
Cyclostome RAR1	inshore hagfish	ACLEILILRICSRYTPEQDTMTFSDGLTLNRTQMHNAGFGPLTDYVFAFAAQLLPLEMDDAEAGLLSAICLICGDRQDLEDPHKVERLQEPLVETLKLYVRRRRPTKPHMFPKMLMKITDLRGISAKGSERVITLKMEIPGSMPPLIQEMLEN
	Japanese lamprey	ACLDILILRICLRYTPEHDTMTFSDGLTLNRTQMHNAGFGPLTDLVFTFAGQLLPLAMDDTEAGLLCAICLITGDRQDLEEPEKVEQLQEPLLEALKVYARRRRPDKPHMFPKILMKITDLRGISAKGSERVITLKMELPGSMPPLIQEMLEN
Cyclostome RAR2	inshore hagfish	ACLDILILRICTRYTPEQDTMTFSDGLTLNRTQMHNAGFGPLTDLVFTFANQLLPLSVDDTEAGLLAAICLISGDRQDLEEPEKVERLQEPLLEAMKVYVRRRRPDKPHMFPKLLMKITDLRGISAKGTERVITLKMEIPGSMPPLIQEMLEN
	Japanese lamprey	ACLDILILRICTRYTPEKDTMTFSDGLTLNRTQMHNAGFGPLTDLVFAFATQLLPLQMNDTEAGLLSAICLICGDRQDLEEPDRVDQLQEPLLEALKLYMRWRRPDKPHMFPKLLMKITDLRGISAKGAERVITLKMEIPGSMPPLIREMLEN
Cyclostome RAR3	inshore hagfish	ACLDILILRICTRYTPEHDTMTFSDGLTLNRTQMHNAGFGPLTDLVFAFAAQLLPLEMDDTEAGLLSSICLITGDRPELEHPERVDRLQEPLLEALKYYVRKRRPTKPHMFPKILMKITDLRGISSKGSDRVITLKMEIPGSMPPLIQEMLEN
	Japanese lamprey	ACLDILILRICTRYTPEHDTMTFSDGLTLNRTQMHNAGFGPLTDLVFAFASQLIPLGMDDTEAGLLSAICLITGDRPELEDPSKVDRLQEPLLEALKFYVRKRRPSKPHMFPKILMKITDLRGISAKGSERVITLKMEIPGSMPPLIQEMLEN
Outgroup	*Ciona intestinalis*	ACLEVLFLRICSRFSPEHDTMTFSDGLTLTRKQMRVCGFGPITEQVFSFAQSLHPLNADATEIGLLSAICLVSADRVDLEEPDKVELLQESLVEGLKYYARKRRPHTPQVFPKLIIKISDLRSISLKGADRVVTVKTEIPGAMPPLMSEMLEN
	Ciona savignyi	ACLEVLFLRICSRFSPEQDTMTFSDGLTLTRKQMRVCGFGPITEQVFTFAQSLYPLDADAAEIGLLSAICLVSADRPDLEEPDKVEMLQETLVEGLKWYARKRRPNAPQVFPKLIIKISDLRSISLKGADRVVSVKSEIPGAMPPLMSEMLEG
	Polyandrocarpa misakiens	ACLEILFFRICSRYDNSNDTMSFSDGLTLNRDQLRNCAFGPMTEQVFGFAKSLTPFDLDHTEGGLLCAICLMCADRTELEDPEKVEKLQEPLVEGLKWFARKKRPNKPHVFPKMLMKIADLRCIGFKGGDRAMSIRKEMPESMPPLMREMLVD
	Branchiostoma floridae	ACLDILILRLATRLDKESDTVTFINGMMLSRTQMHNAGFGPLTDGVFTFAEGMQKLLFDETEIGLMCSICLVCGDRQGLEDIQRAENLQEPLLEALKAYSRRRIPDDPQRFPKMIMKITDLRSISSKGAERVITLKMELSSPMPPLIAEIWEK
