# Supplementary material for: Evolutionary diversification of retinoic acid receptor ligand-binding pocket structure by molecular tinkering
Source: R Soc Open Sci. 2016 Mar 16;3(3):150484. doi: 10.1098/rsos.150484 (PMC4821253; doi:10.1098/rsos.150484)
Supplement: Supplementary Figure S2. Alignment of the ligand-binding domains (LBDs) of 61 retinoic acid receptor (RAR) sequences retained for ancestral sequence reconstruction. [file rsos150484supp2.pdf]

```
CLUSTAL

Sea_urchin_RAR                   S--KE-CVRNDRNKKKK-K--------NDEV--TESLVIPTEIEDILQEVLRAHRDTFPQ
Acorn_worm_RAR                   S--KE-CVRNDRNKKRK-A--------KHEV--TESLEMNTEMEHIMDIVIKAHIETFPK
Amphioxus_RAR                    S--KE-SVRNDRNKKRK-DKT------QSLEKHTLSYNWTPEIQTIITTVREAHMATLPD
Colonial_ascidian_RAR            L--KE-SVRNDRNKKRG-KEKDGKTGDTSPSGQPEEIVVTPEIENIVSVVAKAHLDTFPK
Pacific_sea_squirt_RAR           L--RE-SVRNDRNKKRG-KEKEGK---SPEQ--NDDVTCSPEIEALVASVYKYHVDTFPL
Vase_tunicate_RAR                L--RE-SVRNDRNKKRG-KEKEG----KSDQNGVDEPSCSPEIEALVTSVHKFHVETFPL
Japanese_lamprey_RAR3            S--KE-SVRNDRSKKKK-EVV------RPEL--LEIPPPCPEIEDLIDRVRRAHQETFPS
Australian_lamprey_RAR3          S--KE-SVRNDRSKKKK-EAV------RPEV--LEVPPPCPEIEDLIDRVRRAHQETFPS
Inshore_hagfish_RAR3             S--KE-SVRNDRSKKKK-DVP------RVEL--VELPPAPPELEDLTSRVCRAHQETFPN
Small-spotted_catshark_RARa      S--KE-SVRNDRNKKKK-DTP------KQEC--SESYTITPETEDLIEKVRKAHQETFPA
Little_Skate_RARa                S--KE-SVRNDRNKKKK-ETP------KQEC--SESYTITPETEDLIEKVRKAHQETFPA
Elephant_Shark_RARa              S--KE-SVRNDRNKKKK-EVP------KQEC--SESYTITPEIEDLIEKVRKAHQETFPA
Japanese_pufferfish_RARab        S--RE-LVRNDRMKKKK-DEK------KPAE--VETYVLSADTEQMIERVRRAHQDTFPS
Zebrafish_RARab                  S--KE-SVRNDRNKRKK-DDK------KQEC--LENYVLSPDTEKMIEQVRKAHQETFPS
Japanese_pufferfish_RARaa        S--KE-SVRNDRNKKKK-DEK------KPEC--IENYVLSPDTEQMINRVRKAHQETFPS
Zebrafish_RARaa                  S--KE-SVRNDRNKKKK-EEK------KPEC--TENYTLSPDTEQMIDRVRKAHQETFPS
Spotted_gar_RARa                 S--KE-SVRNDRNKKKK-DEK------KQEC--TESYVLSPATEDMIAKVRKAHQETFPS
Coelacanth_RARa                  S--KE-SVRNDRNKKKK-DSS------KQEC--SESYIITPETEDLIEKVRKAHQETFPA
Eastern_newt_RARa                S--KE-SVRNDRNKKKKQEAP------KQEC--TESYIITPEVEDLVEKVRKAHQETFPA
African_clawed_frog_RARa         S--KE-SVRNDRNKKKK-ESP------KPEA--IESYILSPETQDLIEKVQKAHQETFPA
Chicken_RARa                     S--KE-SVRNDRNKKKK-DVP------KTEC--SESYIVTPEVEELIEKVRKAHQETFPA
Carolina_anole_RARa              S--KE-SVRNDRNKKKK-DAP------KQEC--SESYIITPEVEDLIEKVRKAHQETFPA
Gray_short-tailed_opossum_RARa S--KE-SVRNDRNKKKK-ETP------KAEC--SESYTLTPEVEELIEKVRKAHQETFPA
Mouse_RARa                       S--KE-SVRNDRNKKKK-EAP------KPEC--SESYTLTPEVGELIEKVRKAHQETFPA
Human_RARa                       S--KE-SVRNDRNKKKK-EVP------KPEC--SESYTLTPEVGELIEKVRKAHQETFPA
Sea_lamprey_RAR2                 ------------------------------------------------------------
Japanese_lamprey_RAR2            S--KE-SVRNDRNKKKK-DVA------KQEP--SETLAMPAEMEVLIEKVHKAHQETFPS
Australian_lamprey_RAR2          S--KE-SVRNDRNKKKK-DVA------KQEP--AETLAMPAEMEVLIEKVRKAHQETFPS
Inshore_hagfish_RAR2             S--KE-SVRNDRNKKKK-DAL------KLEC--MENGSLTPEMEELVEKVRKAHEETFPS
Small-spotted_catshark_RARb      S--KE-SVRNDRNKKKK-EMP------KQEC--SESYTMTAELEDLIEKICKAHQETFPS
Little_Skate_RARb                S--KE-SVRNDRNKKKK-ETP------KQEC--SESYTMTAELENLIERIRKAHQETFPS
Elephant_Shark_RARb              S--KE-SVRNDRNKKKK-EAP------KQEC--SESYTMTAELEDLIEKICKAHQETFPS
Spotted_gar_RARb                 S--KE-SVRNDRNKKKK-EVP------KAEC--TESYELTAELEDLIAKIRKAHQETFPS
Japanese_pufferfish_RARb         S--PS-AVRNDRNKKKK-ESP------KPEL--AESYELTAELETIIEKIRKAHQETFPS
Coelacanth_RARb                  ------AVRNDRNKKKK-ETP------KQED--AESYEMTAELDDLIEKIRKAHQETFPS
Eastern_newt_RARb                S--KE-SVRNDRNKKKK-EPI------KQEC--IESLEMTAELDDLTEKIRKAHQETFPS
Western_clawed_frog_RARb         S--KE-SVRNDRNKKKK-EPS------KIEC--IENYEMTAELDDLTEKIRKAHQETFPS
Chicken_RARb                     S--KE-SVRNDRNKKKK-EPT------KQES--TENYEMTAELDDLTEKIRKAHQETFPS
Carolina_anole_RARb              S--KE-SVRNDRNKKKK-EPL------KQEF--MENYEMTAELDDLTEKIRKSHQETFPS
Gray_short-tailed_opossum_RARb S--KE-SVRNDKNKKKK-EPS------KQEF--TESYEMTAELDDLTEKIRKAHQETFPS
Mouse_RARb                       S--KE-SVRNDRNKKKK-EPS------KQEC--TESYEMTAELDDLTEKIRKAHQETFPS
Human_RARb                       S--KE-SVRNDRNKKKK-ETS------KQEC--TESYEMTAELDDLTEKIRKAHQETFPS
Australian_lamprey_RAR1          S--KE-AVRNDRNKKRK-EVV---VVARSPEVPPERYELSCEQEQLVDKIRKSHQETFPS
Japanese_lamprey_RAR1            S--KE-AVRNDRNKKRK-EAPVVVAAVRSPEVPPERYELSCEQEQLVDKIRKSHQETFPS
Sea_lamprey_RAR1                 S--KE-AVRNDRNKKRK-EVV-----ARSPEIVPRSYELSPEQEQMVDKIRCAHQETFPS
Inshore_hagfish_RAR1             S--KEAAVRNDRNKKKK-EPP------KSPEVEVECYTLSPELEDMINKINKAHQETFPS
spotted_catshark_RARg            S--KE-SVRNDRNKRK--PV-------KEEV--PERYEVTPEVEGLIEKVCRAHQQTCPS
Little_skate_RARg                S--KE-SVRNDRNVKRK-SP-------DDEL--VSGVEMTSELERLIERVCRAHRETFPS
Elephant_shark_RARg              S--KE-SVRNDRNKKK--EL-------KEEA--AESLPVAAETEDLIQKVCRAHQETFPS
Japanese_pufferfish_RARgb        S--KE-AVRNDRNKKKK-DV-------KEEVVLPENYELSGELEELVNKVSKAHQETFPS
Zebrafish_RARgb                  S--KE-AVRNDRNKKKK-DV-------KEEVVLPESYELSGELEELVNKVSKAHRETFPS
Japanese_pufferfish_RARga        SIFNT-AVRNDRNKKKK-DV-------KEEVVLPESYELSGELEELVNKVSKAHQETFPS
Zebrafish_RARga                  S--KE-AVRNDRNKKKK-DV-------KDEVIPPESYELSGELEELVNKVSKAHQETFPS
Spotted_gar_RARg                 S--KE-AVRNDRNKKKK-DV-------KEEVVVPESYELSSELEELVSKVSKAHQETFPS
Coelacanth_RARg                  S--KE-AVRNDRNKKKK-EV-------KEEVV-SESYEMTPELEELIQKVSKAHQETFPS
African_clawed_frog_RARg         S--KE-AVRNDRNKKKK-EI-------KEEVVLPDSYEMPPEMEELIQKVSKAHQETFPS
Western_clawed_frog_RARg         S--KE-AVRNDRNKKKK-EI-------KEEVVVPDSYEMPPEMEELIQKVSKAHQETFPS
Eastern_Newt_RARg                S--KE-AVRNDRNKKKK-EI-------KEEVV-TDSYEMPPEMEALIQKVSKAHQETFPS
Chicken_RARg                     S--KE-AVRNDRNKKKK-EV-------KEEA--ADSPELSPALEELIQKVSRAHQETFPS
Mouse_RARg                       S--KE-AVRNDRNKKKK-EV-------KEEGS-PDSYELSPQLEELITKVSKAHQETFPS
Human_RARg                       S--KE-AVRNDRNKKKK-EV-------KEEGS-PDSYELSPQLEELITKVSKAHQETFPS
```

```
Sea_urchin_RAR                    RPLHPIHNADDEEEGVPNHPVS-------NFKGQDIDMVMFDYVTDMSSRAIVMVVDFAK
Acorn_worm_RAR                    DTPKPGFKTGSD-----------------------TDLLMFQYVTDMSSRAIIMVVEFAK
Amphioxus_RAR                     MGKLPKYKVKNA-----------------AEQRGPTDIELWQHFSDLCTETIIKIVQFAK
Colonial_ascidian_RAR             NEDLNKYNTAEGQ---GDKPLSPPTPSDASGSPPPVDVKLWSKFSDLSTKSIIKIVEFAK
Pacific_sea_squirt_RAR            NSNLRKYKIASP-----------VATT--ELETKKTDSNLWEKFAELSTKCIVKIVEFAK
Vase_tunicate_RAR                 SSELKKYQIPSP-----------PIV---KDTSAKTDSNLWEKFAELSTKCIVKIVEFAK
Japanese_lamprey_RAR3             LCQLGKYTTSNG-----------------SGQRATLDANLWDKFSELSTKCIVKTVEFAK
Australian_lamprey_RAR3           LCQLGKYTTSNG-----------------SGQRVTLDANLWDKFSELSTKCIVKTVEFAK
Inshore_hagfish_RAR3              LCQLGKYTMTNG-----------------SGQRVTLDADLWDKFSDLSTKCIVRTVEFAK
Small-spotted_catshark_RARa       LCQLGKYTTNNS-----------------AEQRVTLDIDLWDKFSELSTKCIIKTVEFAK
Little_Skate_RARa                 LCQLGKYTTNNS-----------------AEKRVTLDIDLWDKFSELSTKCIIKTVEFAK
Elephant_Shark_RARa               LCQLGKYTTNNS-----------------AEQRVTLDIDLWDKFSELSTKCIIKTVEFAK
Japanese_pufferfish_RARab         LCQLGKYTTSNS-----------------SEHRVSLDVSLWDKFSELSTKCIIKTVEFAK
Zebrafish_RARab                   LCQLGKYTTNNS-----------------ADHRVALDVDLWDKFSELSTKCIIKTVEFAK
Japanese_pufferfish_RARaa         LCQLGKYTTTNS-----------------SERRVALDVDLWDKFSELSTKCIIKTVEFAK
Zebrafish_RARaa                   LCQLGKYTTSNS-----------------SERRVALDVDLWDKFSELSTKCIIKTVEFAK
Spotted_gar_RARa                  LCQLGKYTTNNS-----------------SDQRVSLDIDLWDKFSELSTKCIIKTVEFAK
Coelacanth_RARa                   LCQLGKYTTNNS-----------------SDQRVSLDIDLWDKFSELSTKCIIKTVEFAK
Eastern_newt_RARa                 LCQLGKYTTNNS-----------------SEERVSLDIDLWDKFSELSTKCIIKTVEFAK
African_clawed_frog_RARa          LCQLGKYTTSFS-----------------SEQRVSLDIDLWDKFSELSTKCIIKTVEFAK
Chicken_RARa                      LCQLGKYTTNNS-----------------SEQRVSLDIDLWDKFSELSTKCIIKTVEFAK
Carolina_anole_RARa               LCQLGKYTTNNS-----------------SDQRVSLDIDLWDKFSELSTKCIIKTVEFAK
Gray_short-tailed_opossum_RARa    LCQLGKYTTNNS-----------------SEQRVSLDIDLWDKFSELSTKCIIKTVEFAK
Mouse_RARa                        LCQLGKYTTNNS-----------------SEQRVSLDIDLWDKFSELSTKCIIKTVEFAK
Human_RARa                        LCQLGKYTTNNS-----------------SEQRVSLDIDLWDKFSELSTKCIIKTVEFAK
Sea_lamprey_RAR2                  ---------NSS-----------------ADHRVQLDLGLWDKFSELATKCIIKIVEFAK
Japanese_lamprey_RAR2             LYQLGKYTMNSS-----------------ADHRVQLDLGLWDKFSELATKCIIKIVEFAK
Australian_lamprey_RAR2           LYQLGKYTMNSS-----------------ADHRVQLDLGLWDKLSELATKCIIKIVEFAK
Inshore_hagfish_RAR2              LYQLGKYTMHSS-----------------TEHRVQLDLSLWDKFSELATKCIVKIVEFAK
Small-spotted_catshark_RARb       LCQLGKYTTNSS-----------------ADHRVRLDLGLWDKFSELATKCIIKIVEFAK
Little_Skate_RARb                 LCQLGKYTTNSS-----------------ADHRVRLDLGLWDKFSELATKCIIKIVEFAK
Elephant_Shark_RARb               LCQLGKYTTNSS-----------------ADHRVRLDLGLWDKFSELATKCIIKIVEFAK
Spotted_gar_RARb                  LCQLGKYTTNSS-----------------ADHRVRLDLGLWDKFSELATKCIIKIVEFAK
Japanese_pufferfish_RARb          LCQLGKYTTNSS-----------------ADHRVRLDLGLWDKFSELATKCIIKIVEFAK
Coelacanth_RARb                   LCQLGKYTTNSS-----------------ADHRVRLDLGLWDKFSELATKCIIKIVEFAK
Eastern_newt_RARb                 LCQLGKYTTNSS-----------------AEQRIRLDLGLWDKFSELATKCIIKIVEFAK
Western_clawed_frog_RARb          LCQLGKYTTNSS-----------------AEQRVRLDLGLWDKFSELATKCIIKIVEFAK
Chicken_RARb                      LCQLGKYTTNSS-----------------ADHRVRLDLGLWDKFSELATKCIIKIVEFAK
Carolina_anole_RARb               LCQLGKYTTNSS-----------------ADHRVRLDLGLWDKFSELATKCIIKIVEFAK
Gray_short-tailed_opossum_RARb    LCQLGKYTTNSS-----------------ADHRVRLDLGLWDKFSELATKCIIKIVEFAK
Mouse_RARb                        LCQLGKYTTNSS-----------------ADHRVRLDLGLWDKFSELATKCIIKIVEFAK
Human_RARb                        LCQLGKYTTNSS-----------------ADHRVRLDLGLWDKFSELATKCIIKIVEFAK
Australian_lamprey_RAR1           LCQLGKYTTNSC-----------------ADHRVQLDLGLWDKFSELSTKCIVKIVEFAK
Japanese_lamprey_RAR1             LCQLGKYTTNSC-----------------ADHRVQLDLGLWDKFSELSTKCIVKIVEFAK
Sea_lamprey_RAR1                  LCQLGKYTTNSC-----------------ADHRVQLDLGLWDKFSELSTKCIVKIVEFAK
Inshore_hagfish_RAR1              LCQLGKYTTNSS-----------------AEHRVQLDLNLWDKFSELSTKCIVKIVEFAK
spotted_catshark_RARg             LCQLGKYTTNSS-----------------ADHRVQLDLGLWDKFSELATKCIIKIVEFAK
Little_skate_RARg                 LCQLGKYTTNSS-----------------ADHRVQLDLGLWDKFSELATKCIIKI-----
Elephant_shark_RARg               LCQLGKYTTNSS-----------------ADHRVQLDLGLWDKFSELSTKCIIKIVEFAK
Japanese_pufferfish_RARgb         LCQLGKYTTNSS-----------------SDHRVQLDLGLWDKFSELSTKCIIKIVEFAK
Zebrafish_RARgb                   LCQLGKYTTNSS-----------------ADHRVQLDLGLWDKFSELSTKCIIKIVEFAK
Japanese_pufferfish_RARga         LGQLGKYTTNSS-----------------SDHRVQLDLGLWDKFSELSTKCIIKIVEFAK
Zebrafish_RARga                   LCQLGKYTTNSS-----------------SDHRIQLDLGLWDKFSELSTKCIIKIVEFAK
Spotted_gar_RARg                  LCQLGKYTTNSS-----------------ADHRVQLDLGLWDKFSELSTKCIIKIVEFAK
Coelacanth_RARg                   LCQLGKYTTNSS-----------------ADHRVQLDLGLWDKFSELATKCIIKIVEFAK
African_clawed_frog_RARg          LCQLGKYTTNSS-----------------ADQRVQLDLGLWDKFSELSTKCIIKIVEFAK
Western_clawed_frog_RARg          LCQLGKYTTNSS-----------------ADHRVQLDLGLWDKFSELSTKCIIKIVEFAK
Eastern_Newt_RARg                 LCQLGKYTTNSS-----------------ADHRVQLDLGLWHKFSELATKCIIKIVEFAK
Chicken_RARg                      LCQLGKYTMSSS-----------------AQHRVQLDPGLWDKFSELATKCIIKIVEFAK
Mouse_RARg                        LCQLGKYTTNSS-----------------ADHRVQLDLGLWDKFSELATKCIIKIVEFAK
Human_RARg                        LCQLGKYTTNSS-----------------ADHRVQLDLGLWDKFSELATKCIIKIVEFAK
```

```
Sea_urchin_RAR                 KLPGFLSLSTQDQITLLKASCLDIMILRICSRFNPQDASVTFTTGLTLTQGQLKAGGFGS
Acorn_worm_RAR                 KLPGFLQLTTPDQITLLKAACLEIMVLRISYRFNRDDGSVTFTSGLTLSNKQLKTGGFGT
Amphioxus_RAR                  KVPGFTTFGTADQITLLKAACLDILILRLATRLDKESDTVTFINGMMLSRTQMHNAGFGP
Colonial_ascidian_RAR          SVPGFTDLTIADQITLLKSACLEILFFRICSRYDNSNDTMSFSDGLTLNRDQLRNCAFGP
Pacific_sea_squirt_RAR         GVPGFQDFTIADQITLLKCACLEVLFLRICSRFSPEQDTMTFSDGLTLTRKQMRVCGFGP
Vase_tunicate_RAR              GIPGFQDFTIADQITLLKCACLEVLFLRICSRFSPEHDTMTFSDGLTLTRKQMRVCGFGP
Japanese_lamprey_RAR3          RIPGFSTLGIDDQITLLKAACLDILILRICTRYTPEHDTMTFSDGLTLNRTQMHNAGFGP
Australian_lamprey_RAR3        RIPGFSTLGIDDQITLLKAACLDILILRICTRYTPEHDTMTFSDGLTLNRTQMHNAGFGP
Inshore_hagfish_RAR3           RIPGFSTLAIDDQITLLKAACLDILILRICTRYTPEHDTMTFSDGLTLNRTQMHNAGFGP
Small-spotted_catshark_RARa    HLPGFTTLTIADQITLLKAACLDILILRICTRYTPDQDTMTFSDGLTLNRTQMHNAGFGP
Little_Skate_RARa              HLPGFTTLTIADQITLLKAACLDILILRICTRYTQDQDTMTFSDGLTLNRTQMHNAGFGP
Elephant_Shark_RARa            HLPGFTTLTIADQITLLKAACLDILILRICTRYTPDQDTMTFSDGLTLNRTQMHNAGFGP
Japanese_pufferfish_RARab      QLPGFTTLTIADQITLLKAACLDILILRICTRYTPDQDTMTFSDGLTLNRTQMHNAGFGP
Zebrafish_RARab                QLPGFTTLTIADQITLLKAACLDILILRICTRYTPDQDTMTFSDGLTLNRTQMHNAGFGP
Japanese_pufferfish_RARaa      QLPGFVTLTIADQITLLKAACLDILILRICTRYTPEQDTMTFSDGLTLNRTQMHNAGFGP
Zebrafish_RARaa                QLPGFTTLTIADQITLLKAACLDILILRICTRYTPEQDTMTFSDGLTLNRTQMHNAGFGP
Spotted_gar_RARa               QLPGFTTLTIADQITLLKAACLDILILRICTRYTPEQDTMTFSDGLTLNRTQMHNAGFGP
Coelacanth_RARa                QLPGFTTLTIADQITLLKAACLDILILRICTRYTPEQDTMTFSDGLTLNRTQMHNAGFGP
Eastern_newt_RARa              QLPGFTTLTIADQITLLKAACLDILILRICTRYTPDQDTMTFSDGLTLNRTQMHNAGFGP
African_clawed_frog_RARa       QLPGFTTLTIADQITLLKSACLDILILRICTRYTPDQDTMTFSDGLTLNRTQMHNAGFGP
Chicken_RARa                   QLPGFTTLTIADQITLLKAACLDILILRICTRYTPEQDTMTFSDGLTLNRTQMHNAGFGP
Carolina_anole_RARa            QLPGFTTLTIADQITLLKAACLDILILRICTRYTPDQDTMTFSDGLTLNRTQMHNAGFGP
Gray_short-tailed_opossum_RARa QLPGFTTLTIADQITLLKAACLDILILRIC-RYTPEQDTMTFSDGLTLNRTQMHNAGFGP
Mouse_RARa                     QLPGFTTLTIADQITLLKAACLDILILRICTRYTPEQDTMTFSDGLTLNRTQMHNAGFGP
Human_RARa                     QLPGFTTLTIADQITLLKAACLDILILRICTRYTPEQDTMTFSDGLTLNRTQMHNAGFGP
Sea_lamprey_RAR2               RLPGFVNLSIADQITLLKAACLDILILRICSRYTPEQDTMTFSDGLTLNRTQMHNAGFGP
Japanese_lamprey_RAR2          RLPGFVNLSIADQITLLKAACLDILILRICTRYTPEQDTMTFSDGLTLNRTQMHNAGFGP
Australian_lamprey_RAR2        RLPGFVNLSIADQITLLKAACLDILILRICTRYTPEQDTMTFSDGLTLNRTQMHNAGFGP
Inshore_hagfish_RAR2           HLPGFVGLSIADQITLLKAACLDILILRICTRYTPEKDTMTFSDGLTLNRTQMHNAGFGP
Small-spotted_catshark_RARb    RLPGFTSLTIADQITLLKAACLDILILRICTRYTPEQDTMTFSDGLTLNRIQMHNAGFGP
Little_Skate_RARb              RLPGFTSLTIADQITLLKAACLDILILRICTRYTPEQDTMTFSDGLTLNRTQMHNAGFGP
Elephant_Shark_RARb            RLPGFTSLTIADQITLLKAACLDILILRICTRYTPDQDTMTFSDGLTLNRTQMHNAGFGP
Spotted_gar_RARb               RVPGFTGLTIADQITLLKAACLDILILRICTRYTPDQDTMTFSDGLTLNRTQMHNAGFGP
Japanese_pufferfish_RARb       RVPGFTALTIADQITLLKAACLDILILRICTRYTPDQDTMTFSDGLTLNRTQMHNAGFGP
Coelacanth_RARb                RVPGFTSLTIADQITLLKAACLDILILRICTRYTPEQDTMTFSDGLTLNRTQMHNAGFGP
Eastern_newt_RARb              RLPGFTSLTIADQITLLKAACLDILILRICTRYTPEQDTMTFSDGLTLNRTQMHNAGFGP
Western_clawed_frog_RARb       RLPGFTSLTIADQITLLKAACLDILILRICTRYTPEQDTMTFSDGLTLNRTQMHNAGFGP
Chicken_RARb                   RLPGFTSLTIADQITLLKAACLDILILRICTRYTPEQDTMTFSDGLTLNRTQMHNAGFGP
Carolina_anole_RARb            RLPGFTSLTIADQITLLKAACLDILILRICTRYTPEQDTMTFSDGLTLNRTQMHNAGFGP
Gray_short-tailed_opossum_RARb RLPGFTSLTIADQITLLKAACLDILILRICTRYTPEQDTMTFSDGLTLNRTQMHNAGFGP
Mouse_RARb                     RLPGFTGLTIADQITLLKAACLDILILRICTRYTPEQDTMTFSDGLTLNRTQMHNAGFGP
Human_RARb                     RLPGFTGLTIADQITLLKAACLDILILRICTRYTPEQDTMTFSDGLTLNRTQMHNAGFGP
Australian_lamprey_RAR1        RLPGFPSLTIADQITLLKAACLEILILRICTRHTPEQDTMTFSDGLTLNRTQMHNAGFGP
Japanese_lamprey_RAR1          RLPGFPSLTIADQITLLKAACLEILILRICSRYTPEQDTMTFSDGLTLNRTQMHNAGFGP
Sea_lamprey_RAR1               RMPGFPSLTIADQITLLKAACLDILILRICSRYTPEQDTMTFSDGLTLNRTQMHNAGFGP
Inshore_hagfish_RAR1           RLPGFPSLTIADQITLLKAACLDILILRICLRYTPEHDTMTFSDGLTLNRTQMHNAGFGP
spotted_catshark_RARg          RLPGFTSLTIADQITLLKAACLDILMLRICIRYTPDQDTMTFSDGLTLNRTQMHNAGFGP
Little_skate_RARg              ------------------------------------------------------------
Elephant_shark_RARg            RLPGFTGLTIADQITLLKAACLDILMLRICTRYTPDQDTMTFSDGLTLNRTQMHNAGFGP
Japanese_pufferfish_RARgb      RLPGFTTLTIADQITLLKSACLDILMLRICTRYTPEQDTMTFSDGLTLNRTQMHNAGFGP
Zebrafish_RARgb                RLPGFTSLTIADQITLLKSACLDILMLRICTRYTPEQDTMTFSDGLTLNRTQMHNAGFGP
Japanese_pufferfish_RARga      RLPGFTSLTIADQITLLKSACLDILMLRICTRYTPEQDTMTFSDGLTLNRTQMHNAGFGP
Zebrafish_RARga                RLPGFTTLTIADQITLLKSACLDILMLRICTRYTPEQDTMTFSDGLTLNRTQMHNAGFGP
Spotted_gar_RARg               RLPGFTTLTIADQITLLKAACLDILMLRICTRYTPEQDTMTFSDGLTLNRTQMHNAGFGP
Coelacanth_RARg                RLPGFTTLTIADQITLLKAACLDI------------------------------------
African_clawed_frog_RARg       RLPGFTTLTIADQITLLKSACLDILMLRICTRYTPEQDTMTFSDGLTLNRTQMHNAGFGP
Western_clawed_frog_RARg       RLPGFTTLTIADQITLLKSACLDILMLRICTRYTPEQDTMTFSDGLTLNRTQMHNAGFGP
Eastern_Newt_RARg              RLPGFATLTIADQITLLKAACLDILMLRICTRYTPEQDTMTFSDGLTLNRTQMHNAGFGP
Chicken_RARg                   RLPGFTALSIADQITLLKAACLDILMLRICTRYTLEQDTMTFSDGLTLNRTQMHNAGFGP
Mouse_RARg                     RLPGFTGLSIADQITLLKAACLDILMLRICTRYTPEQDTMTFSDGLTLNRTQMHNAGFGP
Human_RARg                     RLPGFTGLSIADQITLLKAACLDILMLRICTRYTPEQDTMTFSDGLTLNRTQMHNAGFGP
```

```
Sea_urchin_RAR                  LLDVIFTFASSLSRMHIDETEIALLSAICLISE--DRTGLEDAPRIEKMQEPLLEGLRLY
Acorn_worm_RAR                  LMDTIGQFAQQISSLNLDDTELALLSAICLISG--DRSGLEEPLKIEKMQEPLLEALRIY
Amphioxus_RAR                   LTDGVFTFAEGMQKLLFDETEIGLMCSICLVCG--DRQGLEDIQRAENLQEPLLEALKAY
Colonial_ascidian_RAR           MTEQVFGFAKSLTPFDLDHTEGGLLCAICLMCA--DRTELEDPEKVEKLQEPLVEGLKWF
Pacific_sea_squirt_RAR          ITEQVFTFAQSLYPLDADAAEIGLLSAICLVSA--DRPDLEEPDKVEMLQETLVEGLKWY
Vase_tunicate_RAR               ITEQVFSFAQSLHPLNADATEIGLLSAICLVSA--DRVDLEEPDKVELLQESLVEGLKYY
Japanese_lamprey_RAR3           LTDLVFAFAAQLLPLEMDDTEAGLLSSICLITG--DRPELEHPERVDRLQEPLLEALKYY
Australian_lamprey_RAR3         LTDLVFAFAAQLLPLEMDDTEAGLLSSICLITG--DRPELEHPERVDRLQEPLLEALKFY
Inshore_hagfish_RAR3            LTDLVFAFASQLIPLGMDDTEAGLLSAICLITG--DRPELEDPSKVDRLQEPLLEALKFY
Small-spotted_catshark_RARa     LTDLVFAFANQLLPLEMDDAETGLLSAICLICG--DRQDLEEPEKVDKLQEPLLEALKIY
Little_Skate_RARa               LTDLVFAFANQLLPLEMDDAETGLLSAICLICG--DRQDLEEPEKVDKLQEPLLEALKIY
Elephant_Shark_RARa             LTDLVFAFANQLLPLEMDDAETGLLSAICLICG--DRQDLEQAEKVDKLQEPLLEALKIY
Japanese_pufferfish_RARab       LTDLVFSFASQLLPLEMDDAETGLLSAICLLCG--DRQDLQESDKVDILQEPIVEALKMY
Zebrafish_RARab                 LTDLVFAFANQLLPLEMDDAETGLLSAICLLCG--DRQDLEQSDKVDELQEPLLEALKIY
Japanese_pufferfish_RARaa       LTDLVFAFANQLLPLEMDDAETGLLSAICLLCGETDRQDLEQAEKVDILQEPLLEALKIY
Zebrafish_RARaa                 LTDLVFAFANQLLPLEMDDAETGLLSAICLLCG--DRQDLEQADKVDVLQEPLLEALKIY
Spotted_gar_RARa                LTDLVFAFANQLLPLEMDDAETGLLSAICLLCG--DRQDLEQSDRVDTLQEPLLEALKVY
Coelacanth_RARa                 LTDLVFAFANQLLPLEMDDAETGLLSAICLICG--DRQDLEQPDKVDKLQEPLLEALKIY
Eastern_newt_RARa               LTDLVFAFANQLLPLEMDDAETGLLSAICLICG--DRQDLEQPDKVDKLQEPLLEALKIY
African_clawed_frog_RARa        LTDLVFAFANQPVPLEMDDAETGLLSAICLICG--DRQDLEQPDKVDKLQEPLLEALKIY
Chicken_RARa                    LTDLVFAFANQLLPLEMDDAETGLLSAICLICG--DRQDLEQPDKVDKLQEPLLEALKIY
Carolina_anole_RARa             LTDLVFAFANQLLPLEMDDAETGLLSAICLICG--DRQDLEQPDRVDKLQEPLLEALKIY
Gray_short-tailed_opossum_RARa LTDLVFAFANQLLPLEMDDAETGLLSAICLICG--DRQDLEQPDKVDTLQEPLLEALKVY
Mouse_RARa                      LTDLVFAFANQLLPLEMDDAETGLLSAICLICG--DRQDLEQPDKVDMLQEPLLEALKVY
Human_RARa                      LTDLVFAFANQLLPLEMDDAETGLLSAICLICG--DRQDLEQPDRVDMLQEPLLEALKVY
Sea_lamprey_RAR2                LTDLVFAFANQLLPLSVDDTEAGLLAAICLMCA--DRQDLEEPEKVERLQEPLLEAMKVY
Japanese_lamprey_RAR2           LTDLVFTFANQLLPLSVDDTEAGLLAAICLISG--DRQDLEEPEKVERLQEPLLEAMKVY
Australian_lamprey_RAR2         LTDLVFAFANQLLPLSVDDTEAGLLAAICLISG--DRQDLEEPEKVERLQEPLLEAMKVY
Inshore_hagfish_RAR2            LTDLVFAFATQLLPLQMNDTEAGLLSAICLICG--DRQDLEEPDRVDQLQEPLLEALKLY
Small-spotted_catshark_RARb     LTDLVFTFANQLLPLEMDDTETGLLSAICLICG--DRQDLEDAEKVDKLQEPLLEALKIY
Little_Skate_RARb               LTDLVFTFAKQLLPLEMDDTETGLLSAICLICG--DRQDLEDAGKVDKLQEPLLEALKIY
Elephant_Shark_RARb             LTDLVFTFANQLLPLEMDDTETGLLSAICLICG--DRQDLEDAGKVDKLQEPLLEALKIY
Spotted_gar_RARb                LTDLVFTFANQLLPLEMDDTETGLLSAICLISG--DRQDLEEPAKVDKLQEPLLEALKIY
Japanese_pufferfish_RARb        LTDLVFTFANQLLPIEMDDTETGLLSAICLISG--DRQDLEEPSKVDQLQEPLLEALKIY
Coelacanth_RARb                 LTDLVFTFANQLLPLEMDDTESGLLSAICLICG--DRQDLEEPTKVDKLQEPLLEALKIY
Eastern_newt_RARb               LSDLGFPFANQLLPLEMDDTQTGHLSAICLICE--DRQDLEEPTKVDKLQEPLLEALKIY
Western_clawed_frog_RARb        LTDLVFTFANQLLPLEMDDTETGLLSAICLICE--DRQDLEEPAKVDKLQEPLLEALKIY
Chicken_RARb                    LTDLVFTFANQLLPLEMDDTETGLLSAICLICG--DRQDLEEPMKVDKLQEPLLEALKIY
Carolina_anole_RARb             LTDLVFTFANQLLPLEMDDTETGLLSAICLICG--DRQDLEEPAKVDELQEPLLEALKIY
Gray_short-tailed_opossum_RARb LTDLVFTFANQLLPLEMDDTETGLLSAICLICG--DRQDLEEPTKVDKLQEPLLEALKIY
Mouse_RARb                      LTDLVFTFANQLLPLEMDDTETGLLSAICLICG--DRQDLEEPTKVDKLQEPLLEALKIY
Human_RARb                      LTDLVFTFANQLLPLEMDDTETGLLSAICLICG--DRQDLEEPTKVDKLQEPLLEALKIY
Australian_lamprey_RAR1         LTDYVFAFAAQLLPLEMDDAEAGLLSAICLICG--DRQDLEDPHKVERLQEPLVETLKLY
Japanese_lamprey_RAR1           LTDYVFAFAAQLLPLEMDDAEAGLLSAICLICG--DRQDLEDPHKVERLQEPLVETLKLY
Sea_lamprey_RAR1                LTDLVFAFASQLLPLEMDDTEAGLLSAICLICG--DRQDLEEPQKVERLQEPLLEALKLY
Inshore_hagfish_RAR1            LTDLVFTFAGQLLPLAMDDTEAGLLCAICLITG--DRQDLEEPEKVEQLQEPLLEALKVY
spotted_catshark_RARg           LTDLLFAFANRLLPLEMDDTETGLLCAICLICG--DRMDLEEPEKVDQLQEPLLEALKVY
Little_skate_RARg               ------------------------------------------------------------
Elephant_shark_RARg             LTDL--------------------------------------------------------
Japanese_pufferfish_RARgb       LTDLVFAFAGQLLPLEMDDTETGLLSAICLICG--DRMDLEEPEKVDKLQEPLLEALKIY
Zebrafish_RARgb                 LTDLVFAFAGQLLPLEMDDTETGLLSAICLICG--DRMDLEEPHRVDQLQEPLLEALKIY
Japanese_pufferfish_RARga       LTDLVFAFAGQLLPLEMDDTETGLLSAICLICG--DRMDLEEPEKVDKLQEPLLEALKIY
Zebrafish_RARga                 LTDLVFAFAGQLLPLEMDDTETGLLSAICLICG--DRMDLEEPERVDRLQEPLLEALKIY
Spotted_gar_RARg                LTDLVFAFAGQLLPLEMDDTETGLLSAICLICG--DRMDLEEPQKVDRLQEPLLEALKLY
Coelacanth_RARg                 -----------------------------LICR--DRMDLEEPEKVDKLQEPLLEALKIY
African_clawed_frog_RARg        LTDLVFSFADQLLPLEMDDTETGLLSAICLICG--DRMDLEEPEKVEKLQEPLLEALKFY
Western_clawed_frog_RARg        LTDLVFAFADQLLPLEMDDTETGLLSAICLICG--DRMDLEEPEKVEKLQEPLLEALKFY
Eastern_Newt_RARg               LTDLVFAFAEQLLPLEMDDTETGLLSAICLICG--DRMDLEEPEKVDKLQEPLLEALKIY
Chicken_RARg                    LTDLVFAFAGQLLPLEMDDTETGLLSAICLICG--DRQDLEEPEQVERLQEPLLEALRVY
Mouse_RARg                      LTDLVFAFAGQLLPLEMDDTETGLLSAICLICG--DRMDLEEPEKVDKLQEPLLEALRLY
Human_RARg                      LTDLVFAFAGQLLPLEMDDTETGLLSAICLICG--DRMDLEEPEKVDKLQEPLLEALRLY
```

```
Sea_urchin_RAR                 VRKRRPKESHFFAKLLMKITDLRCISVK---SAEKVFDMKVEFV-KEMPALISEMIDKND
Acorn_worm_RAR                 VRRRRPTEPHAFAKILMKITDLRSISVK---GAERVLHLKLQIP-VDMPQIIQEMVESDE
Amphioxus_RAR                  SRRRIPDDPQRFPKMIMKITDLRSISSK---GAERVITLKMELS-SPMPPLIAEIWEKQN
Colonial_ascidian_RAR          ARKKRPNKPHVFPKMLMKIADLRCIGFK---GGDRAMSIRKEMPKESMPPLMREMLVDED
Pacific_sea_squirt_RAR         ARKRRPNAPQVFPKLIIKISDLRSISLK---GADRVVSVKSEIPSGAMPPLMSEMLEGDE
Vase_tunicate_RAR              ARKRRPHTPQVFPKLIIKISDLRSISLK---GADRVVTVKTEIPCGAMPPLMSEMLENDE
Japanese_lamprey_RAR3          VRKRRPTKPHMFPKILMKITDLRGISSK---GSDRVITLKMEIP-GSMPPLIQEMLENPD
Australian_lamprey_RAR3        VRKRRPTKPHMFPKILMKITDLRGISSK---GSDRVITLKMEIP-GSMPPLIQEMLENPD
Inshore_hagfish_RAR3           VRKRRPSKPHMFPKILMKITDLRGISAK---GSERVITLKMEIP-GSMPPLIQEMLENSD
Small-spotted_catshark_RARa    VRKRRPNKPHMFPKMLMKITDLRSISAK---GAERVITLKMEIP-GSMPPLIQEMLENSD
Little_Skate_RARa              VRKRRPNKPHMFPKMLMKITDLRSISAK---GAERVITLKMEIP-GSMPPLIQEMLENSD
Elephant_Shark_RARa            VRKRRPNKPHMFPKMLMKITDLRSISAK---GAERVITLKMEIP-GSMPPLIQEMLENSD
Japanese_pufferfish_RARab      VRRRRPEKPCMFPKILMKITDLRSISVK---GAERVITLKMEIP-GSMPPLIQEMLENSD
Zebrafish_RARab                VRNRRPHKPHMFPKMLMKITDLRSISAK---GAERVITLKMEIP-GSMPPLIQEMLENSE
Japanese_pufferfish_RARaa      VRRRRPHKPHMFPKMLMKITDLRSISAK---GAERVITLKMEIP-GSMPPLIQEMLENSE
Zebrafish_RARaa                VRNRRPHKPHMFPKMLMKITDLRSISAK---GAERVITLKMEIP-GSMPPLIQEMLENSE
Spotted_gar_RARa               VRKRRPHKPHMFPKMLMKITDLRSISAK---GAERVITLKMEIP-GSMPPLIQEMLENSE
Coelacanth_RARa                VRKRRPNKPHMFPKMLMKITDLRSISAK---GAERVITLKMEIP-GSMPPLIQEMLENSE
Eastern_newt_RARa              VRKRRPNKPHMFPKMLMKITDLRSISAK---GAERVITLKMEIP-GSMPPLIQEMLENSE
African_clawed_frog_RARa       VRTRRPQKPHMFPKMLMKITDLRTVSAK---GAERVITLKMEIP-GAM-PLIQEMLENSE
Chicken_RARa                   VRKRRPNKPHMFPKMLMKITDLRSISAK---GAERVITLKMEIP-GSMPPLIQEMLENSE
Carolina_anole_RARa            VRKRRPSKPHMFPKMLMKITDLRSISAK---GAERVITLKMEIP-GSMPPLIQEMLENSE
Gray_short-tailed_opossum_RARa VRKRRPSRPHMFPKMLMKITDLRSISAK---GAERVITLKMEIP-GSMPPLIQEMLENSE
Mouse_RARa                     VRKRRPSRPHMFPKMLMKITDLRSISAK---GAERVITLKMEIP-GSMPPLIQEMLENSE
Human_RARa                     VRKRRPSRPHMFPKMLMKITDLRSISAK---GAERVITLKMEIP-GSMPPLIQEMLENSE
Sea_lamprey_RAR2               VRRRRPDKPHMFPKLLMKITDLRGISAK---GMERVITLKMEIP-GSMPPLIQEMLENSY
Japanese_lamprey_RAR2          VRRRRPDKPHMFPKLLMKITDLRGISAK---GTERVITLKMEIP-GSMPPLIQEMLENSY
Australian_lamprey_RAR2        VRRRRPDKPHMFPKLLMKITDLRGISAK---GAERVITLKMEIP-GSMPPLIQEMLENSD
Inshore_hagfish_RAR2           MRWRRPDKPHMFPKLLMKITDLRGISAK---GAERVITLKMEIP-GSMPPLIREMLENSD
Small-spotted_catshark_RARb    IRKRRPNKPHMFPKILMKITDLRSISAK---GAERVITLKLEIP-GSMPPLIQEMLENSE
Little_Skate_RARb              IRKRRPNKPHMFPKILMKITDLRSISAK---GAERVITLKLEIP-GSMPPLIQEMLENSE
Elephant_Shark_RARb            IRKRRPNKPHMFPKILMKITDLRSISAK---GAERVITLKLEIP-GSMPPLIQEMLENSE
Spotted_gar_RARb               IRKRRPSKPHMFPKILMKITDLRSISAK---GAERVITLKMEIP-GSMPPLIQEMLENSE
Japanese_pufferfish_RARb       VRKRRPSKPHMFPKTLMKITDLRSISAK---GAERVISLKMEIP-GSMPPLIQEMLENSE
Coelacanth_RARb                IRKRRPNKPHMFPKILMKITDLRSISAK---GAERVITLKMEIP-GSMPPLIQEMLENSE
Eastern_newt_RARb              IRKRRPSKPHMFPKILMKITDLRSISAK---GAERVITLKLEIP-GSMPPLIQEMLENSE
Western_clawed_frog_RARb       IRKRRPNKPHMFPKILMKITDLRSISAK---GAERVITLKLEIP-GSMPPLIQEMLENSE
Chicken_RARb                   IRKRRPNKPHMFPKILMKITDLRSISAK---GAERVITLKMEIP-GSMPPLIQEMLENSE
Carolina_anole_RARb            IRKRRPNKPHMFPKILMKITDLRSISAK---GAERVITLKLEIP-GSMPPLIQEMLENSE
Gray_short-tailed_opossum_RARb IRKRRPNKPHMFPKILMKITDLRSISAK---GAERVITLKMEIP-GSMPPLIQEMLENSE
Mouse_RARb                     IRKRRPSKPHMFPKILMKITDLRSISAK---GAERVITLKMEIP-GSMPPLIQEMLENSE
Human_RARb                     IRKRRPSKPHMFPKILMKITDLRSISAK---GAERVITLKMEIP-GSMPPLIQEMLENSE
Australian_lamprey_RAR1        VRRRRPTKPHMFPKMLMKITDLRGISAK---G----------------------------
Japanese_lamprey_RAR1          VRRRRPTKPHMFPKMLMKITDLRGISAK---GSERVITLKMEIP-GSMPPLIQEMLENMD
Sea_lamprey_RAR1               VRRRRPSKPHMFPKILMKITDLRGISAK---GSERVITLKMEIP-GSMPPLIQEMLENSI
Inshore_hagfish_RAR1           ARRRRPDKPHMFPKILMKITDLRGISAK---GSERVITLKMELP-GSMPPLIQEMLENSD
spotted_catshark_RARg          ARKRRPQKPYMFPRMLMKITDLRGISAK---GAERAITLKMEIP-GPMPPLIREMLENPD
Little_skate_RARg              ------------------------------------------------------------
Elephant_shark_RARg            ------------------------------------------------------------
Japanese_pufferfish_RARgb      TRRRRPNKPHMFPRMLMKVTDLRGISTK---GAERAITLKTEIP-GPMPPLIREMLENPE
Zebrafish_RARgb                ARRRRPNKPHMFPRMLMKVTDLRGISTK---GAERAITLKMEIP-GPMPPLIREMLENPE
Japanese_pufferfish_RARga      ARRRRPNKPHMFPRMLMKITDLRGISTK---GAERAVTLKTEIP-GPMPPLIREMLENPE
Zebrafish_RARga                ARRRRPNKPHMFPRMLMKITDLRGISTK---GAERAITLKMEIP-GPMPPLIREMLENPE
Spotted_gar_RARg               ARRRRPNKPHMFPRMLMKITDLRGISTK---GAERAITLKMEIP-GPMPPLIREMLENPE
Coelacanth_RARg                ARRRRPSKPHMFPRMLMKITDLRGISTKDYVGAERAITLKMEIP-GPMPPLIREMLENPE
African_clawed_frog_RARg       ARRRRPDKPYMFPRMLMKITDLRGISTK---GAERAITLKLEIP-GPMPPLIREMLENPE
Western_clawed_frog_RARg       ARRRRPDKPYMFPRMLMKITDLRGISTK---GAERAITLKMEIP-GPMPPLIREMLENPE
Eastern_Newt_RARg              ARRRRPNKPYMFPRMLMKITDLRGISTK---GAERAITLKMEIP-GPMPPLIREMLENPE
Chicken_RARg                   ARRRRPSKPHMFPRMLMKITDLRGISTK---GAERAITLKMEIP-GPMPPLIREMLENPE
Mouse_RARg                     ARRRRPSQPYMFPRMLMKITDLRGISTK---GAERAITLKMEIP-GPMPPLIREMLENPE
Human_RARg                     ARRRRPSQPYMFPRMLMKITDLRGISTK---GAERAITLKMEIP-GPMPPLIREMLENPE
```

Sea_urchin_RAR	DE
Acorn_worm_RAR	ES
Amphioxus_RAR	EA
Colonial_ascidian_RAR	TE
Pacific_sea_squirt_RAR	TD
Vase_tunicate_RAR	VE
Japanese_lamprey_RAR3	GP
Australian_lamprey_RAR3	GP
Inshore_hagfish_RAR3	GP
Small-spotted_catshark_RARa	GI
Little_Skate_RARa	GI
Elephant_Shark_RARa	GL
Japanese_pufferfish_RARab	GV
Zebrafish_RARab	GL
Japanese_pufferfish_RARaa	GL
Zebrafish_RARaa	GL
Spotted_gar_RARa	AL
Coelacanth_RARa	GM
Eastern_newt_RARa	GL
African_clawed_frog_RARa	GL
Chicken_RARa	GM
Carolina_anole_RARa	GM
Gray_short-tailed_opossum_RARa	GL
Mouse_RARa	GL
Human_RARa	GL
Sea_lamprey_RAR2	GS
Japanese_lamprey_RAR2	GS
Australian_lamprey_RAR2	GS
Inshore_hagfish_RAR2	GP
Small-spotted_catshark_RARb	GH
Little_Skate_RARb	GH
Elephant_Shark_RARb	GH
Spotted_gar_RARb	GQ
Japanese_pufferfish_RARb	GQ
Coelacanth_RARb	GH
Eastern_newt_RARb	GL
Western_clawed_frog_RARb	GH
Chicken_RARb	GH
Carolina_anole_RARb	GH
Gray_short-tailed_opossum_RARb	GH
Mouse_RARb	GH
Human_RARb	GH
Australian_lamprey_RAR1	--
Japanese_lamprey_RAR1	NA
Sea_lamprey_RAR1	EG
Inshore_hagfish_RAR1	EV
spotted_catshark_RARg	AL
Little_skate_RARg	--
Elephant_shark_RARg	--
Japanese_pufferfish_RARgb	AF
Zebrafish_RARgb	IF
Japanese_pufferfish_RARga	AF
Zebrafish_RARga	AF
Spotted_gar_RARg	AF
Coelacanth_RARg	NF
African_clawed_frog_RARg	AF
Western_clawed_frog_RARg	AF
Eastern_Newt_RARg	AF
Chicken_RARg	MF
Mouse_RARg	MF
Human_RARg	MF
