# Supplementary figures and images for: Evolutionary diversification of retinoic acid receptor ligand-binding pocket structure by molecular tinkering
Source: R Soc Open Sci. 2016 Mar 16;3(3):150484. doi: 10.1098/rsos.150484 (PMC4821253; doi:10.1098/rsos.150484)

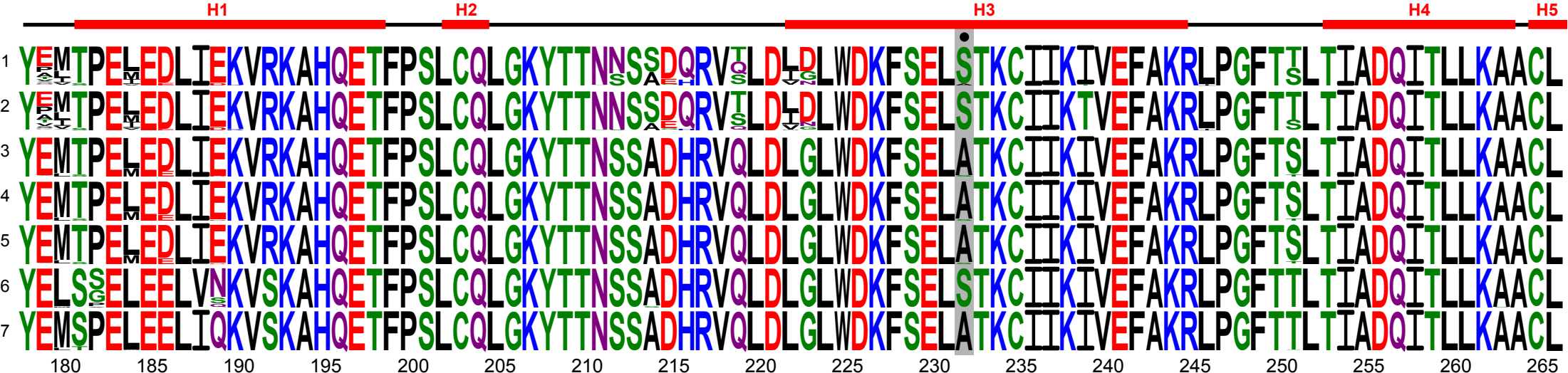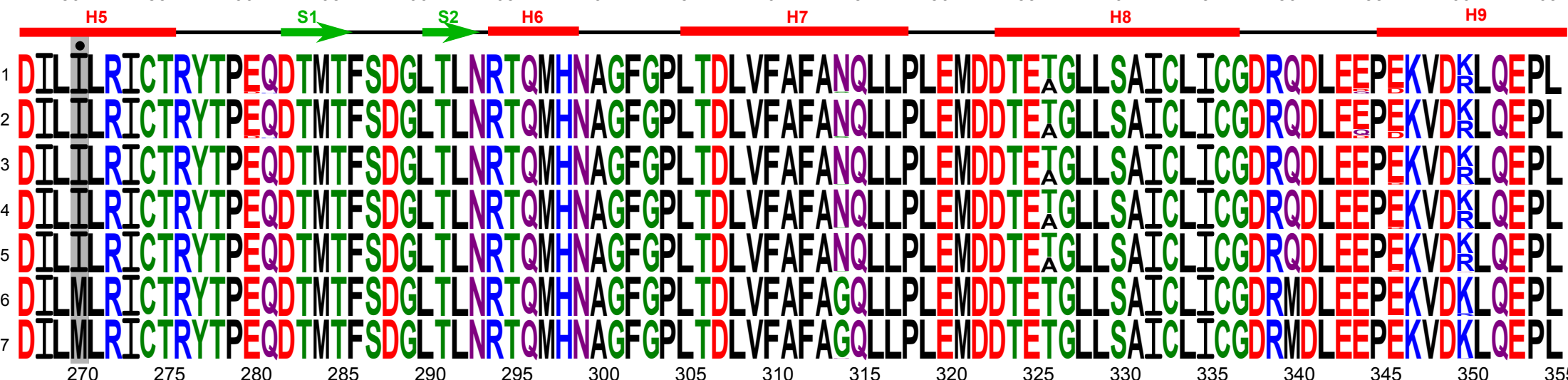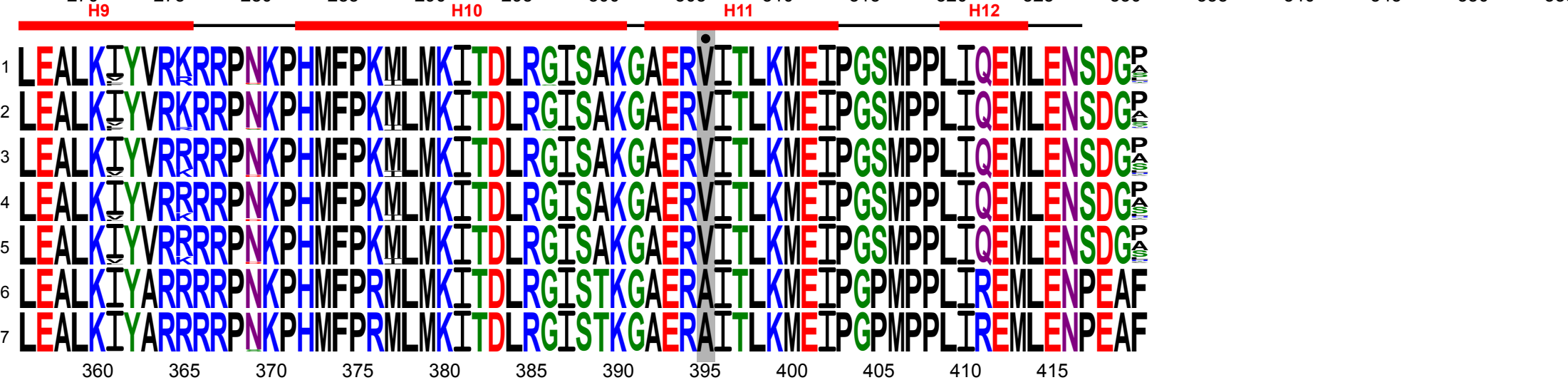

Supplement: Supplementary Figure S3. Ancestral retinoic acid receptor (RAR) sequences calculated at seven nodes of the chordate tree of life. [file rsos150484supp3.pdf]
